# EphrinB2-mediated CDK5/ISL1 pathway enhances cardiac lymphangiogenesis and alleviates ischemic injury by resolving post-MI inflammation

**DOI:** 10.1038/s41392-024-02019-4

**Published:** 2024-11-18

**Authors:** Yingnan Bai, Liming Chen, Fanghao Guo, Jinghong Zhang, Jinlin Hu, Xuefei Tao, Qing Lu, Wenyi Li, Xueying Chen, Ting Gong, Nan Qiu, Yawei Jin, Lifan Yang, Yu Lei, Chengchao Ruan, Qing Jing, John P. Cooke, Shijun Wang, Yunzeng Zou, Junbo Ge

**Affiliations:** 1 Department of Cardiology, Shanghai Institute of Cardiovascular Diseases, Zhongshan Hospital and Institute of Biomedical Sciences, Fudan University, Shanghai, China; 2 Center for Reproductive Medicine & Fertility Preservation Program, International Peace Maternity and Child Health Hospital, School of Medicine, Shanghai Jiao Tong University, Shanghai, China; 3 CAS Key Laboratory of Tissue Microenvironment and Tumor, Shanghai Institute of Nutrition and Health, Innovation Center for Intervention of Chronic Disease and Promotion of Health, University of Chinese Academy of Sciences, Chinese Academy of Sciences, Shanghai, China; 4 Department of Cardiovascular Surgery, Guangdong Provincial Hospital of Chinese Medicine, The Second Affiliated Hospital of Guangzhou University of Chinese Medicine, The Second Clinical College of Guangzhou University of Chinese Medicine, Guangzhou, Guangdong China; 5 Department of Geriatric Cardiology, Sichuan Provincial People’s Hospital, University of Electronic Science and Technology of China, Chengdu, Sichuan China; 6 Department of Radiology, Shanghai Dongfang Hospital, Shanghai Tongji University School of Medicine, Shanghai, China; 7 Department of Endocrinology, Tongren Hospital, Shanghai JiaoMo Tong University School of Medicine, Shanghai, China; 8 Department of Physiology and Pathophysiology, Shanghai Key Laboratory of Bioactive Small Molecules, State Key Laboratory of Medical Neurobiology, School of Basic Medical Sciences, Fudan University, Shanghai, China; 9 Department of Cardiovascular Sciences, Houston Methodist Research Institute, 6670 Bertner Ave., Houston, TX USA; 10 Minhang Hospital, Fudan University, Shanghai, China; 11 Institute of Advanced Medicine, Henan University, Kaifeng, Henan China

**Keywords:** Cardiology, Lymphangiogenesis

## Abstract

EphrinB2 (erythropoietin-producing hepatoma interactor B2) is a key Eph/ephrin family member, promoting angiogenesis, vasculogenesis, and lymphangiogenesis during embryonic development. However, the role of EphrinB2 in cardiac lymphangiogenesis following myocardial infarction (MI) and the potential molecular mechanism remains to be demonstrated. This study revealed that EphrinB2 prevented ischemic heart post-MI from remodeling and dysfunction by activating the cardiac lymphangiogenesis signaling pathway. Deletion of EphrinB2 impaired cardiac lymphangiogenesis and aggravated adverse cardiac remodeling and ventricular dysfunction post-MI. At the same time, overexpression of EphrinB2 stimulated cardiac lymphangiogenesis which facilitated cardiac infiltrating macrophage drainage and reduced inflammation in the ischemic heart. The beneficial effects of EphrinB2 on improving clearance of inflammatory response and cardiac function were abolished in *Lyve1* knockout mice. Mechanistically, EphrinB2 accelerated cell cycling and lymphatic endothelial cell proliferation and migration by activating CDK5 and CDK5-dependent ISL1 nuclear translocation. EphrinB2 enhanced the transcriptional activity of ISL1 at the VEGFR3 *(FLT4*) promoter, and VEGFR3 inhibitor MAZ51 significantly diminished the EphrinB2-mediated lymphangiogenesis and deteriorated the ischemic cardiac function. We uncovered a novel mechanism of EphrinB2-driven cardiac lymphangiogenesis in improving myocardial remodeling and function after MI.

## Introduction

Ischemic heart disease is a leading cause of morbidity and mortality worldwide. Many survivors of acute myocardial infarction (MI) will progress toward post-MI heart failure (HF), characterized by pathological cardiac remodeling and reduced systolic function. Despite recent advances in percutaneous coronary intervention (PCI) and pharmacological therapies, the prognosis for patients with post-MI HF remains unfavorable. Cardiac ischemic injury leads to cardiomyocyte death and fibrosis, accompanied by myocardial edema and inflammation^1^. It has been demonstrated that, in addition to a significant number of cardiomyocyte deaths, there is a high incidence of non-cardiomyocyte deaths, including those of endothelial cells and fibroblasts, in myocardial biopsies of heart failure patients. This finding indicates that endothelial cells play a crucial role in maintaining and supporting cardiac blood and energy supply under hypoxic conditions by promoting angiogenesis. Many protective interventions have targeted the blood vasculature system, rather than the lymphatic system of the heart, for treating ischemic heart disease. A limited understanding of lymphangiogenesis regulation and the absence of timely interventions targeting the lymphatic system may contribute to pathological remodeling following MI. Under physiological conditions, the cardiac lymphatic system returns extravasated macromolecules and fluids to the systemic circulation while modulating immune responses^2,3^. Cardiac lymphatic insufficiency leads to myocardial edema, inflammation, and dysfunction under pathological conditions^2–5^. The selective stimulation of cardiac lymphangiogenesis has emerged as a promising approach to mitigate cardiac dysfunction after MI. For example, it has been observed that stimulation of cardiac lymphangiogenesis was associated with improved cardiac function after experimentally induced MI^2–4^. Although VEGF-C/VEGFR-3 signaling is a well-known and classical signaling pathway leading to the activation of lymphatic vessels^2^, we hypothesized that alternative pathways may also be involved in promoting cardiac lymphangiogenesis to restore heart function after acute MI.

Erythropoietin-producing hepatoma interactors (Ephrins) are transmembrane ligands for erythropoietin-producing hepatoma receptors (Ephs) tyrosine kinases. The mammalian Ephrins/Ephs system comprises eight cell surface-anchored ephrin ligands and fourteen receptor tyrosine kinases. EphrinB2 (erythropoietin-producing hepatoma interactor B2) is an important guidance molecule in vascular development and postnatal angiogenesis^6,7^. Furthermore, EphrinB2 is implicated in developing lymphatic vessels and in the maintenance of lymphatic function^2,8^. The interaction of EphrinB2 and its cognate receptor EphB4 maintains the integrity of lymphatic endothelial cells (LECs) to decrease permeability and lymphoedema^9^. Blockade of EphrinB2 caused lymphatic valve defects^10^. Deletion of the specific intracellular domain of EphrinB2 in knock-in mice substantially impaired vasculogenesis, angiogenesis, and lymphangiogenesis during the development^11,12^. EphrinB2-mediated signaling has been considered a target in microvascular growth and maturation^13^. Endothelial cell-specific expression of EphrinB2 was shown to induce angiogenesis in the ischemic area in a model of hindlimb ischemia^14^. The systemic delivery of the fusion protein (EphrinB2-Fc) resulted in selective stimulation of myocardial angiogenesis following experimental MI^15^. However, the role of EphrinB2 in regulating cardiac lymphangiogenesis in the pathological remodeling process after MI, and the underlying mechanisms remain unexplored.

Using an unbiased RNA sequencing (RNA-seq) approach, we identified LIM homeodomain transcription factor Islet1 (ISL1) as a downstream factor of EphrinB2 signaling that promotes cardiac lymphangiogenesis. ISL1, a transcription factor highly expressed in cardiac progenitor cells, has been reported to contribute to enhanced vascularization and reduced myocardial fibrosis after intramyocardial transfer to the infarct and border zone of infarcted hearts^16^. Furthermore, cardiovascular progenitor cells (CPCs) expressing ISL1 (ISL1-CPCs) spheroids have the potential to differentiate into endothelial cells and form new blood vessels, facilitating cardiac repair and recovery from MI^17^. This work was followed by Maruyama et al., who identified ISL1-expressing progenitor cells as a potential non-venous origin of cardiac LECs using genetic lineage tracing approaches^18^. In view of the unique role of ISL1 in cardiac lymphatic vessel development, we speculated that ISL1 may also play an essential role in cardiac lymphangiogenesis through its transcriptional regulation of LECs in pathological conditions.

In the present work, we identified a critical role of EphrinB2 in cardiac lymphangiogenesis in the pathological remodeling process after acute MI. Our data revealed that knockout of EphrinB2 significantly suppressed cardiac lymphangiogenesis after MI, while overexpression of EphrinB2 increased cardiac lymphangiogenesis, accelerated the resolution of inflammation, and improved cardiac remodeling and dysfunction. Mechanistically, we demonstrated that EphrinB2 activated CDK5 under hypoxic conditions, and thereby promoted ISL1 nuclear translocation through a CDK5/ISL1 interaction, and this process contributed to the EphrinB2-driven lymphangiogenic process. In vivo, overexpression of EphrinB2 markedly reduced the F4/80^+^Ly6C^high^ macrophages in the infarct zone, however, this effect was attenuated in *Lyve1* knockout (*Lyve1*^−/−^) mice with EphrinB2 overexpression. *Lyve1*, the lymphatic marker gene, has been demonstrated to induce the docking and transit of immune cells through the lymphatic endothelium, and this process has been shown to promote the clearance of cardiac infiltrating inflammatory macrophages and cardiac recovery. Therefore, the diminished cardioprotective impact of EphrinB2 on *Lyve1*^−/−^mice suggests a crucial role of EphrinB2-mediated cardiac lymphangiogenesis in the resolution of inflammation after MI. Moreover, VEGFR3 inhibitor MAZ51 significantly diminished the EphrinB2-mediated lymphangiogenesis and impaired the heart repair post-MI, further confirming that cardiac lymphangiogenesis has an impact on myocardial remodeling and dysfunction. In conclusion, our findings suggest that targeting EphrinB2 and cardiac lymphangiogenesis may confer new therapeutic benefits in the treatment of ischemic heart disease.

## Results

### A key role for EphrinB2 in regulating cardiac lymphangiogenesis post-acute myocardial infarction (MI)

**Fig. 1:**
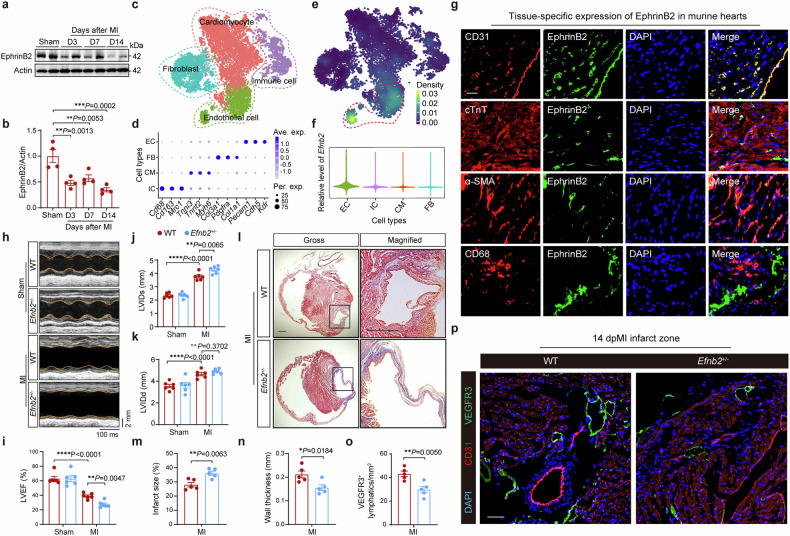
A key role of EphrinB2 in cardiac lymphangiogenesis after acute myocardial infarction (AMI). a Representative immunoblotting images showing EphrinB2 protein levels in the hearts of wild-type (WT) mice subjected to sham or MI operation (at 3, 7, and 14 days after operation). **b** Quantification of **a** normalized to Actin and presented relative to the sham group (n = 4 per group). **c** t-distributed stochastic neighbor embedding (t-SNE) plot showing the cardiac cells isolated from murine hearts that were clustered into four cell populations using the single-cell RNA sequencing data (GSE120064). Colors indicate different cell populations. **d** Dot plot showing feature genes for the four cell populations. **e** Feature plot showing the transcriptional expression of *Efnb2* in each cell. Colors denote the relative expression of *Efnb2*. **f** Violin plot showing the transcriptional expression of *Efnb2* across the four cell populations. **g** Representative immunofluorescence staining images showing myocardium co-stained by EphrinB2 (green) and DAPI (blue) with CD31 (red), cTnT (red), αSMA (red) and CD68 (red), respectively. Scar bar: 20 μm. **h** Representative M-mode echocardiographic images showing the cardiac function of *Efnb2*^*+/−*^ mice and their WT littermates after sham or MI operation. The yellow lines indicate the endocardium of the anterior and posterior walls at mid-papillary muscle level. **i**–**k** Quantification of echocardiographic parameters in **h** (LVEF, LVIDs, and LVIDd, n = 6 per group). **l** Representative histological images showing infarct and fibrosis area of *Efnb2*^*+/−*^ mice and their WT littermates after MI operation assessed by Masson Trichrome staining. Magnified views of black boxes are shown in the right lane. Scar bar: 1 mm. **m**, **n** Quantification of infarct size of myocardium and wall thickness of the infarct area (n = 5 per group). **o** Quantification of **p** (n = 5 per group). **(p)** Representative immunofluorescence images showing myocardium co-stained by CD31 (red), VEGFR3 (green), and DAPI (blue) of *Efnb2*^*+/−*^ mice and their WT littermates after MI operation. Scar bar: 50 μm. *P < 0.05, **P < 0.01, ***P < 0.001, ***P < 0.0001. **b** by Kruskal–Wallis with Dunn test, **i**–**k** by one-way ANOVA with Tukey posthoc test, and **m**–**o** by unpaired Student’s test. D days, ave. exp. average expression, per. exp., percent expresse, LVEF left ventricularejection fraction, LVIDs left ventricular internal dimension during systole, LVIDd left ventricular internal dimension during diastole, DAPI 4’,6-diamidino-2-phenylindole, and TUNEL terminal deoxynucleotidyl transferase-mediated dUTP-biotin nick end labeling

EphrinB2 is a highly conserved protein in mammals, that is crucial for angiogenic growth and the expansion of vascular and lymphatic systems. We hypothesized that an EphrinB2-mediated pathway may be involved in promoting cardiac lymphangiogenesis to restore cardiac function after acute MI. Accordingly, to assess the transcriptional changes of the *Efnb2* gene (encoding EphrinB2) in the murine hearts post-MI, we analyzed the microarray data (GSE6580) from the Gene Expression Omnibus (GEO) database and found that the normalized expression of the *Efnb2* gene was downregulated at 3, 5, 7, and 14 days post-MI (supplementary Fig. 1a). Consistently, a significant decline of EphrinB2 protein level was observed in the ischemic area of hearts at 3 days after MI, and EphrinB2 further decreased at 2 weeks after MI (Fig. 1a, b). Next, we explored the *Efnb2* expression profile in different cell populations in the heart using the single-cell RNA sequencing (scRNA-seq) data obtained from murine hearts. After dimensionality reduction and cell annotation, four major cell populations, including cardiomyocytes (CMs), endothelial cells (ECs), fibroblasts (FBs), and immune cells (ICs) were identified from the scRNA-seq data (Fig. 1c, d). Intriguingly, enrichment of *Efnb2* expression was observed in the EC cluster (Fig. 1e, f). To verify the cardiac localization of EphrinB2 in vivo, we performed anti-EphrinB2 immunofluorescence staining. We confirmed the intense expression of EphrinB2 in the EC cluster, as indicated by the co-localization of EphrinB2 with CD31 (Fig. 1g). We further assessed the distribution of EphrinB2 in EC subtypes in the scRNA-seq data (supplementary Fig 1b, c). We found that EphrinB2 is highly expressed in three EC subtypes including arterial endothelial, endocardial, and lymphatic ECs (LECs) (supplementary Fig 1d). To further characterize the localization of EphinB2 in lymphatics, we performed immunofluorescence staining in the myocardium and observed enrichment of EphrinB2 in lymphatic vessels double-labeled with VEGFR3 and LYVE1 (supplementary Fig 1e). To further explore the role of EphrinB2 in cardiac lymphangiogenesis and remodeling post-MI, we generated *Efnb2* gene knockout mice (supplementary Fig 2a). However, the homozygous *Efnb2*-null mice suffered early postnatal death (supplementary Fig 2b). Accordingly, we used the heterozygous *Efnb2*^+/−^ mice in this study. The knockdown efficacy of *Efnb2* in the mouse heart was confirmed by Western blotting, and reduced expression of EphrinB2 was observed in *Efnb2*^+/−^ mice (supplementary Fig 2c, d). Then, *Efnb2*^+/−^ mice and their wild-type (WT) littermates were subjected to MI or sham operation and followed up for 2 weeks. Compared with WT littermates, *Efnb2*^+/−^ mice had a higher mortality rate after MI (supplementary Fig 3a). Two weeks after MI, the cardiac left ventricular (LV) function was assessed by echocardiography (Fig. 1h). *Efnb2*^+/−^ mice manifested a stunning decline in LV ejection fraction (LVEF) (Fig. 1i). Meanwhile, the LV internal dimension during systole (LVIDs) was significantly increased in *Efnb2*^+/−^ mice compared to their WT counterparts (Fig. 1j, k). Consistently, histological analysis showed that *Efnb2*^+/−^ mice displayed a larger infarct size and thinner wall of the infarct areas after 2 weeks of MI (Fig. 1l–n). Since EphrinB2 is enriched in LECs, we further questioned whether EphrinB2 deficiency impaired the formation of cardiac lymphatic vessels. As expected, the VEGFR3^+^ lymphatic vessels were reduced significantly (Fig. 1o, p). In addition, cell apoptosis in the post-MI heart was significantly increased in *Efnb2*^+/−^ mice (supplementary Fig 3b, c). These data indicate an important role of EphrinB2 in promoting lymphangiogenesis and improving cardiac remodeling and function after MI.

### EphrinB2 is essential for improved cardiac recovery and remodeling post-MI

Considering that global knockout of EphrinB2 is lethal, and organ failure other than the heart can also lead to cardiac damage and dysfunction, we therefore further explored the impact of shRNA-mediated knockdown of EphrinB2 on cardiac function and remodeling post-MI. Mice were transfected by adeno-associated virus-mediated *Efnb2* shRNA (AAV-sh*Efnb2*) via tail-vein injection and cardiac inhibition of EphrinB2 was confirmed by Western blotting (supplementary Fig 4a). Both AAV-sh*Efnb2* and AAV-control-shRNA (AAV-shNC) transfected mice were subjected to MI or sham operation (supplementary Fig 5a). After MI for 2 weeks, a significant decline in cardiac function was observed in AAV-sh*Efnb2* mice compared to AAV-shNC controls (supplementary Fig 5b). This was shown by a significantly decreased EF value, and increased LVIDs and LVIDd (supplementary Fig 5c–e), indicating that shRNA-mediated knockdown of EphrinB2 led to a deterioration of LV function and cardiac remodeling after MI. Consistently, we found a thinner LV wall at the infarcted area of AAV-sh*Efnb2* mice accompanied by the enlarged infarct size compared to AAV-shNC mice (supplementary Fig 5f, i, j). In addition, cardiac apoptosis was significantly increased in AAV-sh*Efnb2* mice compared to AAV-shNC mice (supplementary Fig 5g, h).

**Fig. 2:**
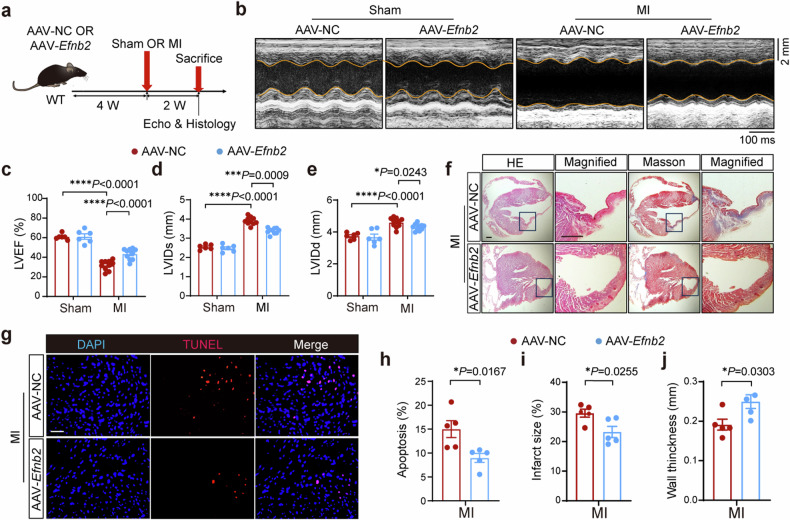
Overexpression of EphrinB2 improved cardiac function post-MI. a Schematic diagram depicting the experimental strategy for EphrinB2 overexpression. **b** Representative M-mode echocardiographic images showing the cardiac function of WT mice that were injected with AAV-NC or AAV-*Efnb2* after sham or MI operation. The yellow lines indicate the endocardium of the anterior and posterior walls at mid-papillary level. **c**–**e** Quantification of echocardiographic parameters in **b** (LVEF, LVIDs, and LVIDd, n = 6-10 per group). **f** Representative histological images showing infarct and fibrosis area of WT mice that were injected with AAV-NC or AAV-*Efnb2* after sham or MI operation assessed by HE and Masson Trichrome staining. Magnified views of black boxes are shown at the right of gross views. Scar bar: 500 μm. **g** Representative TUNEL staining images showing cell apoptosis in the hearts from indicated groups. Scar bar: 50 μm. **h** Quantification of **g** (n = 5 per group). **i**, **j** Quantification of infarct size of the myocardium and wall thickness of the infarct area in **f** (n = 5 per group). *P < 0.05, **P < 0.01, ***P < 0.001, ****P < 0.0001. **c**–**e** by one-way ANOVA with Tukey post-hoc test, and **h**–**j** by unpaired Student’s test. AAV adeno-associated virus serotype, NC negative control, HE hematoxylin and eosin, LVEF left ventricular ejection fraction, LVIDs left ventricular internal dimension during systole, LVIDd left ventricular internal dimension during diastole, DAPI 4’,6-diamidino-2-phenylindole and TUNEL terminal deoxynucleotidyltransferase-mediated dUTP-biotin nick end labeling

We further investigated the possible protective effects of EphrinB2 overexpression in MI. EphrinB2 overexpression in the murine hearts was confirmed by Western blotting (supplementary Fig 4b). Both AAV-*Efnb2* and AAV-NC transfected mice were subjected to MI or sham operation for 2 weeks (Fig. 2a). Intriguingly, we found that AAV-*Efnb2* mice displayed significantly improved cardiac function after MI compared with AAV-NC mice (Fig. 2b). As shown by the echocardiographic analysis, EF value was increased and both LVIDs and LVIDd were decreased in AAV-*Efnb2* mice (Fig. 2c–e). Consistently, EphrinB2 overexpression significantly reduced myocardial infarct size and increased wall thickness (Fig. 2f, i, j). Moreover, EphrinB2 overexpression significantly attenuated cell apoptosis (Fig. 2g, h). These data collectively demonstrated the cardioprotective effects of EphrinB2 post-MI.

### Overexpression of EphrinB2 promotes cardiac lymphangiogenesis and reduces cardiac inflammation post-MI

**Fig. 3:**
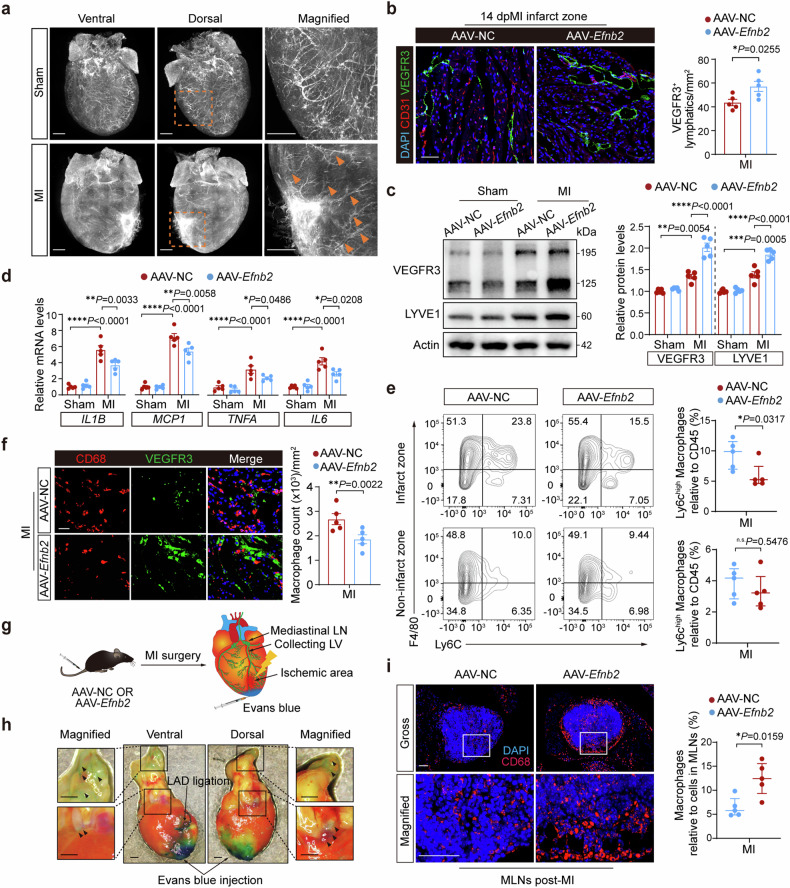
Overexpression of EphrinB2 promotes cardiac lymphangiogenesis and reduces cardiac inflammation post-MI. a Whole mount imaging showing endogenous tdTomato (white) fluorescence in the hearts from *Lyve1*-Cre; *Rosa26*-tdTomato mice after sham or MI operation. Magnified views of yellow dashed boxes are shown in the right lane. The yellow arrows highlight the lymphatics in the infarct area and border zone. Scar bar: 1 mm. **b** (Left) Representative immunofluorescence staining images showing myocardium co-stained by CD31 (red), VEGFR3 (green), and DAPI (blue) of mice injected with AAV-*Efnb2* or AAV-NC after MI operation. Scar bar: 50 μm. (Right) Quantification of VEGFR3^+^ lymphatics (n = 5 per group). **c** (Left) Representative immunoblotting images showing the protein levels of VEGFR3 and LYVE1 in the hearts from indicated groups. (Right) Quantification of immunoblotting results normalized to Actin and presented relative to the AAV-NC group (n = 5 per group). **d** Real-time quantitative reverse transcription polymerase chain reaction (RT-qPCR) analysis determining the mRNA expressions of pro-inflammatory genes in the hearts of mice that were injected with AAV-NC or AAV-*Efnb2* at day 7 post-operation (n = 5 per group). **e** (Left) Fluorescence-activated cell sorter (FACS) analysis of CD45^+^CD11b^+^Ly6G^−^F4/80^+^Ly6C^high^ macrophages in the murine hearts at day 7 post-MI. (Right) Quantification of CD45^+^CD11b^+^Ly6G^−^F4/80^+^Ly6C^high^ macrophages as the percentage of CD45^+^ cells (n = 5 per group). **f** Representative immunofluorescence staining images showing the myocardium co-stained by CD68 (red), VEGFR3 (green), and DAPI (blue) in indicated groups. Scar bar: 20 μm. **g** Schematic diagram depicting the experimental strategy for Evans blue injection from cardiac apex to lymph nodes through the lymphatic system. **h** Representative heart images of Evans blue in the mediastinal lymph nodes and lymphatics. Scar bar: 1 mm. **i** (Left) Representative immunofluorescence staining images showing mediastinal lymph node co-stained by CD68 (red) and DAPI (blue) in indicated groups. Scar bar: 50 μm. (Right) Quantification of CD68^+^ macrophages as the percentage of cells in MLNs. *P < 0.05, **P < 0.01, ***P < 0.001, ****P < 0.0001. **c**, **d** by one-way ANOVA with Tukey post-hoc test, **b** by unpaired Student’s test, and **e** and **i** by Mann–Whitney *U* test. MLN mediastinal lymphatic node, and LV lymphatic vessel

To investigate whether EphrinB2-mediated cardiac lymphangiogenesis contributes to cardioprotective effects via mitigating immune response and inflammation in the context of post-MI injury, we first generated *Lyve1*-Cre; *Rosa26*-tdTomato reporter mice. This allowed us to visualize the cardiac lymphangiogenesis with high clarity using whole-mount fluorescent microscopy. Two weeks after MI, we observed an expanded lymphatic network induced by the endogenous lymphangiogenic response at the border zone in the ischemic mouse heart (Fig. 3a, Supplemental Video 1, 2). After overexpression of *Efnb2* in mice with MI, we found that the density of VEGFR3^+^ lymphatic capillaries was strongly enhanced in AAV-*Efnb2* mice compared to AAV-NC mice (Fig. 3b). Consistent with the potent stimulation of cardiac lymphatic expansion in AAV-*Efnb2* mice, the cardiac protein levels of two lymphatic-selective markers LYVE1 and VEGFR3 were both enhanced by EphrinB2 overexpression (Fig. 3c). These results further clarified the essential role of EphrinB2 in cardiac lymphangiogenesis post-MI.

A growing body of evidence has suggested that inflammatory response can be triggered by MI, and an increase in post-MI inflammation leads to worse cardiac remodeling and dysfunction^19,20^. Cardiac lymphangiogenesis accelerates the clearance of immune cells such as macrophages, thereby reducing cardiac inflammatory injury^21^. In this study, by analyzing the microarray data (GSE775)^22^, we found that M1-type macrophage marker genes, including *Il6*, *Tnfa*, *Il1b*, and *Mcp1*, were increased in the post-MI hearts of mice (supplementary Fig 6a). Our data also indicated the mRNA levels of *Il6*, *Tnfa*, *Il1b*, and *Mcp1* were markedly upregulated after MI. With EphrinB2 overexpression, all these pro-inflammatory cytokines were significantly downregulated after MI (Fig. 3d).

Functionally, Ly6C^high^ and Ly6C^low^ macrophages are similar to M1- and M2-type macrophages in the context of inflammation^17^. Ly6C^high^ proinflammatory monocytes/macrophages migrate into injured tissues, scavenge debris, and secrete a range of pro-inflammatory cytokines, which contribute to ischemic myocardial injury. We sorted CD45^+^CD11b^+^ Ly6G^−^ F4/80^+^ Ly6C^high^ subpopulation by fluorescence-activated cell sorter (FACS) analysis (Supplementary Fig. 7). With EphrinB2 overexpression, we observed a significantly reduced proportion of CD45^+^CD11b^+^ Ly6G^−^ F4/80^+^ Ly6C^high^ macrophages in the infarct zone after 7 days of MI, however, no significant differences in CD45^+^CD11b^+^ Ly6G^−^ F4/80^+^ Ly6C^high^ macrophages in the non-infarct zone were observed between AAV-NC and AAV-*Efnb2* groups (Fig. 3e). Meanwhile, we analyzed microarray data (GSE6580)^23,24^ and found that lymphatic marker *Flt4* (VEGFR3) expression showed negatively correlated trends with the infiltration of cardiac monocytes/macrophages (Supplementary Fig. 6b). Importantly, co-staining of VEGFR3 and CD68 revealed that increased formation of lymphatic capillaries was accompanied by a significant reduction of CD68^+^ cells in AAV-*Efnb2* mice, while accumulation of CD68^+^ macrophages with weak formation of lymphatic capillaries was observed in AAV-NC mice (Fig. 3f). Considering that the decrease in macrophage accumulation in the ischemic zone does not necessarily mean EphrinB2 overexpression-mediated cardiac lymphangiogenesis contributed to immune cell clearance, we determined the drainage of CD68^+^ macrophages in mediastinal lymph nodes (MLNs) after MI (Fig. 3g, h). The proportion of CD68^+^ macrophages in MLNs was significantly increased in AAV-*Efnb2* mice (Fig. 3i). Taken together, our data demonstrate that EphrinB2-driven cardiac lymphangiogenesis augments the resolution of inflammation post-MI.

### *Lyve1* deficiency abrogates the beneficial effects of EphrinB2 overexpression post-MI

**Fig. 4:**
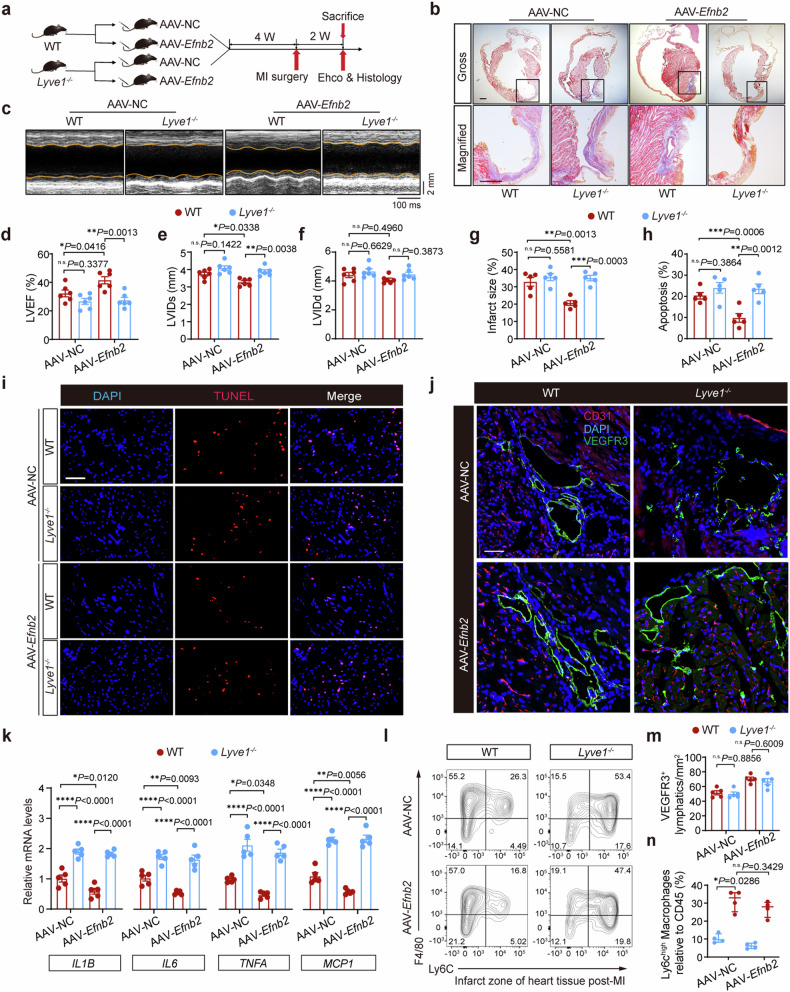
*Lyve1* deficiency abrogates the anti-inflammatory effects of EphrinB2 post-MI. a Schematic diagram depicting the experimental strategy for EphrinB2 overexpression in *Lyve1*^*−/−*^ mice and their WT littermates. **b** Representative histological images from *Lyve1*^*−/−*^ mice and their WT littermates that were injected with AAV-NC or AAV-*Efnb2* assessed by Masson Trichrome staining. Magnified views of black boxes are shown in the bottom lane. Scar bar: 1 mm. **c** Representative M-mode echocardiographic images showing the cardiac function of *Lyve1*^*−/−*^ mice and their WT littermates that were injected with AAV-NC or AAV-*Efnb2*. The yellow lines indicate the endocardium of the anterior and posterior walls at mid-papillary muscle level. **d**–**f** Quantification of echocardiographic parameters in **c** (LVEF, LVIDs, and LVIDd, n = 6 per group). **g** Quantification of infarct size of myocardium in **b** (n = 5 per group). **h** Quantification of apoptotic cells as percentage of all cells in **i**. **i** Representative TUNEL staining images showing cell apoptosis in indicated groups. Scar bar: 50 μm. **j** Representative immunofluorescence staining images showing the myocardium co-stained by CD31 (red), VEGFR3 (green), and DAPI (blue) in indicated groups. Scar bar: 50 μm. **k** Real-time quantitative reverse transcription polymerase chain reaction (RT-qPCR) analysis determining the mRNA expressions of pro-inflammatory genes in the hearts in indicated groups at day 7 post-MI (n = 5 per group). **l** (Left) Fluorescence-activated cell sorter (FACS) analysis of CD45^+^CD11b^+^Ly6G^−^F4/80^+^Ly6C^high^ macrophages in the murine hearts at day 7 post-MI. **m** Quantification of the density of VEGFR3^+^ lymphatics in **j**. **n** Quantification of CD45^+^CD11b^+^Ly6G^−^F4/80^+^Ly6C^high^ macrophages as the percentage of CD45^+^ cells in **l** (n = 4 per group). *P < 0.05, **P < 0.01, ***P < 0.001, ****P < 0.0001. **d–h**, **k**, and **m** by one-way ANOVA with Tukey post-hoc test, and **n** by Mann-Whitney *U* test. LVEF left ventricular ejection fraction, LVIDs left ventricular internal dimension during systole, LVIDd left ventricular internal dimension during diastole, DAPI 4’,6-diamidino-2-phenylindole, HE hematoxylin and eosin, and TUNEL terminal deoxynucleotidyltransferase-mediated dUTPbiotin nick end labeling

To further demonstrate that the resolution of inflammation through EphrinB2-driven cardiac lymphangiogenesis provides an effective strategy to protect hearts against MI, we generated *Lyve1* knockout (*Lyve1*^*−/−*^) mice (Supplementary Fig. 8a). LYVE1 is a hyaluronic acid (HA) receptor that is highly expressed in the cell membrane of LECs, facilitating the adhesion and transit of macrophages under inflammatory conditions^21,25^. Previous studies reported that deletion of *Lyve1* inhibited the docking and transit of leukocytes across the lymphatic endothelium, leading to the exacerbation of chronic inflammation and the long-term decline of cardiac function. Deletion of *Lyve1* was confirmed by Western blotting (Supplementary Fig. 8b, c). Both *Lyve1*^*−/−*^ mice and their WT littermates were injected with AAV-*Efnb2* or AAV-NC vectors for 4 weeks before MI or sham surgery, and cardiac function was assessed at 2 weeks after MI (Fig. 4a). As expected, EphrinB2 overexpression benefited cardiac recovery. However, these cardioprotective effects were not observed in *Lyve1*^*−/−*^ mice, despite the overexpression of EphrinB2, as evidenced by the compromised systolic function and deteriorated cardiac remodeling in AAV9-*Efnb2 Lyve1*^*−/−*^ group compared to AAV9-*Efnb2* WT group (Fig. 4c–f). Consistently, infarct size (Fig. 4b, g) and cardiac apoptosis (Fig. 4h, i) were significantly decreased in WT mice following EphrinB2 overexpression, whereas *Lyve1* deficiency disrupted EphrinB2-mediated cardiac repair. We next examined the density of VEGFR3^+^ lymphatic vessels in the ischemic region of *Lyve1*^−/−^ mice after MI. The normal lymphangiogenic response was observed in *Lyve1*^*−/−*^ mice as the density of VEGFR3^+^ vessels was comparable between *Lyve1*^*−/−*^ and WT mice after MI (Fig. 4j, m). Interestingly, EphrinB2 overexpression failed to reduce MI-induced cardiac inflammation in *Lyve1*^*−/−*^ mice, as cardiac expressions of *Il6*, *Tnfa*, *Il1b*, and *Mcp1* remained higher in *Lyve1*^*−/−*^ mice with AAV-*Efnb2* than those in AAV-*Efnb2* injected WT mice (Fig. 4k). Considering that *Lyve1* is critical for immune cell clearance after MI^21^, we speculated that *Lyve1* deficiency might not affect cardiac lymphangiogenesis, but impaired the clearance of inflammation after acute MI. By FACS analysis, we found that EphrinB2 overexpression significantly reduced CD45^+^CD11b^+^ Ly6G^−^ F4/80^+^ Ly6C^high^ macrophages in the infarct zone in WT mice. By contrast, this subpopulation was comparable between AAV-NC *Lyve1*^*−/−*^ mice and AAV-*Efnb2 Lyve1*^*−/−*^ mice (Fig. 4l, n). These findings collectively suggested that EphrinB2-driven cardiac lymphangiogenesis exerted cardioprotective effects through accelerating pro-inflammatory macrophage drainage and the resolution of inflammation.

### EphrinB2 promotes lymphatic proliferation and migration via the upregulation and nuclear translocation of ISL1

**Fig. 5:**
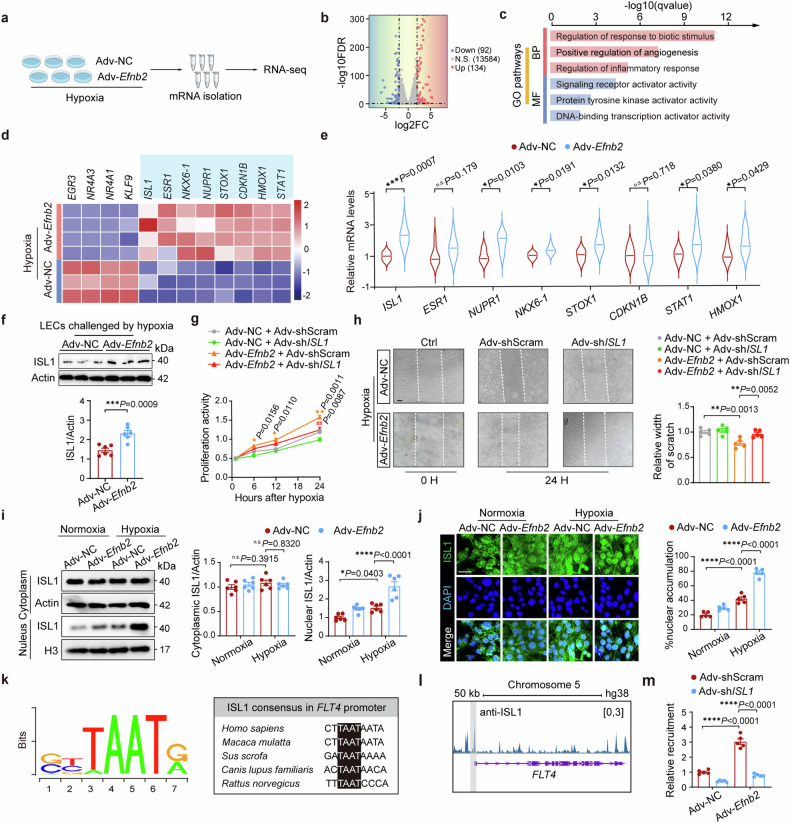
EphrinB2 promoted lymphatic proliferation and migration via the upregulation and nuclear translocation of ISL1. a Schematic diagram depicting the experimental strategy for RNA sequencing. **b** Volcano plot showing significantly differentially expressed genes (DEGs) in lymphatic endothelial cells (LECs) transfected with Adv-NC or Adv-*Efnb2* following RNA sequencing. Genes with log_2_fold-change ≥2.0 and adjusted *P* < 0.05 were considered DEGs. **c** Barplot showing significantly enriched gene ontology (GO) pathways. **d** Heat map showing DEGs involed in endothelial cell proliferation. **e** RT-qPCR analysis determining the mRNA expressions of genes in **d** from LECs transfected with Adv-NC and Adv-*Efnb2* in hypoxic conditions (n = 6 per group). **f** (Top) Representative immunoblotting images showing ISL1 protein levels in LECs treated with Adv-NC or Adv-*Efnb2* under hypoxia. (Bottom) Quantification of the immunoblotting results normalized to Actin (n = 5 per group). **g** Cell proliferation activity of LECs transfected with Adv-NC or Adv-*Efnb2* and Adv-shScram or Adv-sh*ISL1* under hypoxic conditions for indicated times (0, 6, 12, 24 hours) assessed by CCK-8 assay. *(yellow)*P* < 0.05, **(yellow)*P* < 0.01 for Adv-*Efnb2* + Adv-shScram vs Adv-NC + Adv-shScram; ^#^(red)*P* < 0.05, ^##^(red)*P* < 0.01 for Adv-*Efnb2*+Adv-sh*ISL1* vs Adv-*Efnb2* + Adv-shScram. **h** (Left) Wound healing assay determing the effect of *ISL1* knockdown under normoxic or hypoxic conditions for 24 hours. The white dashed lines indicate the terminals of the scratch. Scar bar: 200 μm. (Right) Quantification of results presented relative to normoxia+Adv-shScram group (n = 5 per group). **i** (Left) Representative immunoblotting images showing the nuclear and cytoplasmic extracts of ISL1 in LECs transfected with Adv-NC and Adv-*Efnb2* under normoxic or hypoxic conditions. (Right) Quantification of the immunoblotting results normalized to Actin and presented relative to normoxia + Adv-NC group (n = 6 per group). **j** (Left) Representative immunofluorescence staining images showing LECs co-stained by ISL1 (green) and DAPI (blue) in indicated groups. Scar bar: 20 μm. (Right) Quantification of the immunofluorescence staining intensity in the LEC nuclei presented relative to the Adv-NC group (n = 5 per group). **k** Schematic illustration of conserved *ISL1* consensus located at *FLT4* promoter region across species. **l** Representative ChIP-seq track of ISL1 peaks across the *FLT4* gene. The grey interval indicates the promoter region of *FLT4* gene. **(m)** Chromatin immunoprecipitation (ChIP) assay showing the relative recruitment of ISL1 at *FLT4* promoter region in indicated groups (n = 5 per group). *P < 0.05, **P < 0.01, ***P < 0.001, ****P < 0.0001. **e**, **f** by unpaired Student’s test. **h**–**j,** and **m** by one-way ANOVA with Tukey post-hoc test, and **g** by two-way repeated measurement ANOVA with Bonferroni’s multiple comparisons test. BP biological process, MF molecular function, false discovery rate, and FC fodl change, and hg human genome

To determine the specific role of EphrinB2 in LECs, we conducted in vitro studies by challenging human LECs with hypoxia for 24 hours following transfection with EphrinB2-harboring adenovirus vector (Adv-*Efnb2*) or the negative control vector (Adv-NC). Wound healing analysis showed that the migration ability of LECs was slightly stimulated by hypoxia and potently enhanced by EphrinB2 overexpression (supplementary Fig 9a, b). Meanwhile, the proliferation activity of LECs was detected with Cell Count Kit-8 (CCK-8) assay in time series, which displayed that EphrinB2 overexpression boosted the proliferation activity of LECs in a time-dependent manner under hypoxic conditions (supplementary Fig 9c). To figure out how EphrinB2 promoted lymphatic proliferation and migration, we harvested the LECs transfected with Adv-*Efnb2* or Adv-NC under hypoxia and performed RNA-sequencing analysis (Fig. 5a and supplementary Fig 10a). The volcano plot showed a total of 226 differentially expressed genes (DEGs), with 134 genes upregulated and 92 downregulated after EphrinB2 overexpression (Fig. 5b). Gene Ontology (GO) analysis revealed that positive regulation of angiogenesis and DNA-binding transcription activity, which is associated with endothelial cell proliferation, were significantly enriched in Adv-*Efnb2* LECs compared with that in Adv-NC LECs (Fig. 5c). Additionally, we observed that regulation of the cell cycle was activated while immune response was suppressed following EphrinB2 overexpression according to the gene set enrichment analysis (GSEA) (supplementary Fig 10b). The endothelial cell proliferation genes, including *ISL1*, *STAT1*, *STOX1*, *CDKN1B*, *HOMX1*, *ESR1*, *NKX6-1*, and *NUPR1*, were significantly upregulated by EphrinB2 overexpression under hypoxia (Fig. 5d). After analyzing by real-time PCR, we found that Adv-*Efnb2* treatment increased the transcriptional levels of *ISL1*, *NUPR1*, *NKX6-1*, *STOX1*, *STAT1*, and *HMOX1*, but not significantly impacted the mRNA levels of *ESR1* and *CDKN1B*. Notably, *ISL1* expression showed the most significant increase in hypoxic LECs following EphrinB2 overexpression (Fig. 5e). Next, we examined the protein level of ISL1 in LECs transfected with Adv-*Efnb2* or Adv-NC. Consistent with the transcriptional pattern, ISL1 protein was also largely higher in hypoxia-induced Adv-*Efnb2* LECs than in Adv-NC LECs, while ISL1 protein level did not change by EphrinB2 overexpression under normoxic conditions (Fig. 5f). Attempts were then made to determine whether EphrinB2 promoted lymphatic proliferation and migration via the upregulation of ISL1. Accordingly, we constructed Adv-sh*ISL1* and Adv-shScram, and the knockdown efficiency of Adv-sh*ISL1* against ISL1 was confirmed by Western blotting (supplementary Fig 11a, b). After hypoxia, the contributions of EphrinB2 to facilitate LEC proliferation and migration were markedly abolished following the knockdown of *ISL1* (Fig. 5g, h). In summary, these data confirmed that EphrinB2 overexpression promoted the migration and proliferation of LECs under hypoxic conditions in vitro, due to the upregulation of ISL1 expression in LECs.

Next, we extracted subcellular fractions of ISL1 in LECs. Western blotting analysis revealed that cytoplasmic expression of ISL1 remained unaltered following EphrinB2 overexpression under hypoxia. However, nuclear expression of ISL1 was elevated moderately after hypoxia compared with normoxic conditions, and EphrinB2 overexpression further elevated nuclear ISL1 expression under hypoxia (Fig. 5i). In accordance, immunofluorescence staining revealed that there was more positive immunofluorescence signal of nuclear ISL1 following EphrinB2 overexpression (Fig. 5j), indicating that EphrinB2 overexpression enhanced nuclear translocation of ISL1. To explore the potential transcriptional activity of ISL1 in the nucleus, the matching ISL1 binding motif was identified using the online ISMARA tool^26^, and a highly conserved consensus in *FLT4* promoter was confirmed across species (Fig. 5k). We identified an ISL1-binding site at the *FLT4* promoter region using previously published chromatin immunoprecipitation and sequencing (ChIP-seq) data of the *ISL1* gene in the HepG cell line^26^ (Fig. 5l). Next, we performed a chromatin immunoprecipitation (ChIP) assay using an anti-ISL1 antibody and examined whether the recruitment of ISL1 at the *FLT4* promoter could be affected by EphrinB2 overexpression. The ChIP-qPCR assays displayed that EphrinB2 promoted the recruitment of ISL1 at the *FLT4* promoter in LECs, indicating that EphrinB2 enhanced the binding of ISL1 at *FLT4* promoter and activated the transcriptional activity of ISL1 (Fig. 5m). These data demonstrated that nuclear translocation of ISL1 played a crucial part in driving EphrinB2-mediated lymphangiogenesis.

### EphrinB2 induces nuclear translocation of ISL1 by activating the CDK5 pathway

**Fig. 6:**
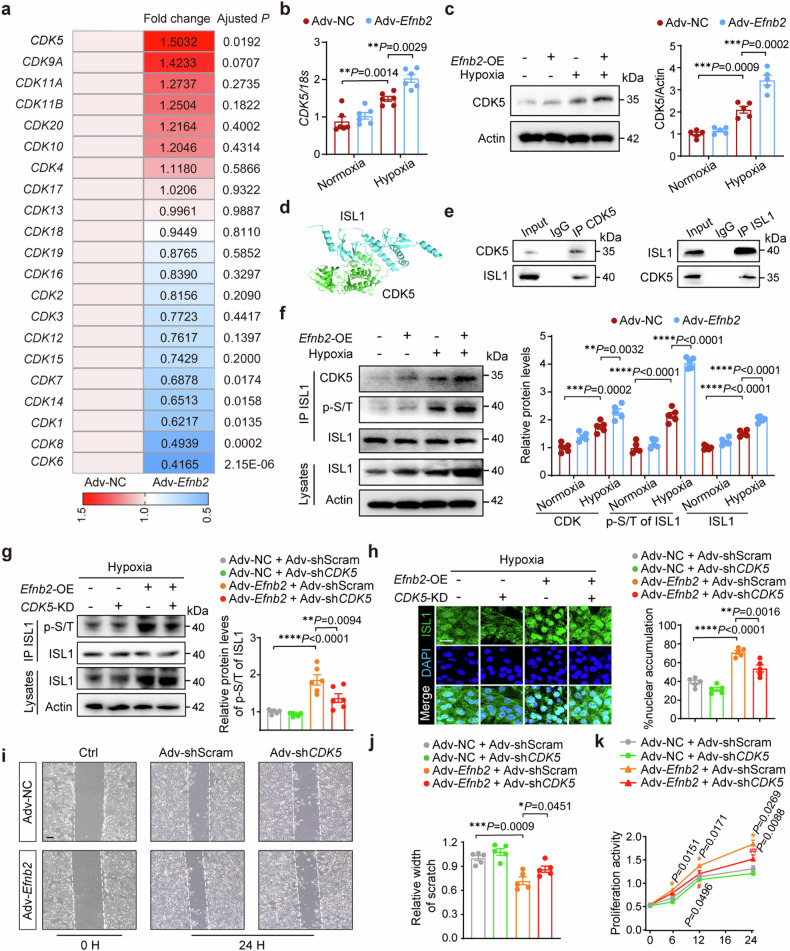
EphrinB2 induced nuclear translocation of ISL1 through activating CDK5 pathway. a Heatmap showing the expressions of *CDKs* in RNA-seq data. **b** RT-qPCR analysis determining the mRNA expression of *CDK5* in LECs transfected with Adv-NC and Adv-*Efnb2* under normoxic or hypoxic conditions (n = 6 per group). **c** (Left) Representative immunoblotting images showing CDK5 protein levels in LECs transfected with Adv-NC or Adv-*Efnb2* under normoxic or hypoxic conditions. (Right) Quantification of the immunoblotting results normalized to Actin and presented relative to normoxia + Adv-NC group (n = 5 per group). **d** A predicted docking model of CKD5 and ISL1 with ClusPro. **e** Co-IP assay was performed to measure the interaction between CDK5 and ISL1. **f** (Left) Representative immunoblotting images showing immunoprecipitation (IP) assay for CDK5 binding to ISL1 and P-S/T levels of ISL1 in LECs transfected with Adv-NC or Adv-*Efnb2* under normoxic or hypoxic conditions. (Right) Quantification of the immunoblotting results normalized to ISL1 or Actin and presented relative to normoxia + Adv-NC group (n = 6 per group). **g** (Left) Representative immunoblotting images showing immunoprecipitation (IP) assay for P-S/T levels of ISL1 in LECs in indicated groups in hypoxic conditions. (Right) Quantification of the immunoblotting results normalized to ISL1 and presented relative to Adv-NC + Adv-shScram group (n = 6 per group). **h** (Left) Representative immunofluorescence images showing LECs co-stained by ISL1 (green) and DAPI (blue) in indicated groups. Scar bar: 20 μm. (Right) Quantification of the immunofluorescence staining intensity in the nuclei presented relative to the Adv-NC + Adv-shScram group (n = 5 per group). **i** Wound healing assay determining th effect of *CDK5* knockdown under hypoxic conditions for 24 h. The white dashed lines indicate the terminals of the scratch. Scar bar: 200 μm. **j** Quantification of **i** presented relative to Adv-NC + Adv-shScram group (n = 5 per group). **k** Cell proliferation activity of LECs with Adv-NC or Adv-*Efnb2* and Adv-shScram or Adv-sh*CDK5* under hypoxic conditions for indicated times (0, 6, 12, 24 hours) assessed by CCK-8 assay. *(yellow)*P* < 0.05, **(yellow)*P* < 0.01 for Adv-*Efnb2* + Adv-shScram vs Adv-NC + Adv-shScram; ^#^(red)*P* < 0.05, ^##^(red)*P* < 0.01 for Adv-*Efnb2*+Adv-sh*CDK5* vs Adv-*Efnb2* + Adv-shScram. *P < 0.05, **P < 0.01, ***P < 0.001, ****P < 0.0001. **b**, **c**, **f**–**h**, and **j** by one-way ANOVA with Tukey post-hoc test, and **k** by two-way repeated measurement ANOVA with Bonferroni’s multiple comparisons test

We next explored how EphrinB2 modulated the nuclear-cytoplasmic shuttling of ISL1. Phosphorylation of ISL1 at serine residues by CDK1 augmented its transcriptional activity and promotes cell proliferation in gastric cancer^27^, indicating the potential involvement of CDKs in regulating ISL1 subcellular localization. Subsequently, we analyzed the mRNA expression of CDKs in our RNA-seq data from hypoxia-induced LECs, revealing the most significant upregulation of CDK5 in EphrinB2 overexpressed LECs (Fig. 6a). Consistent with the RNA-seq findings, EphrinB2 overexpression significantly increased CDK5 mRNA and protein levels in LECs (Fig. 6b, c). We therefore hypothesized that CDK5 could be involved in ISL1 activation in LECs. As expected, a predicted docking model between CDK5 and ISL1 was confirmed with the ClusPro tool, which means a possibility of physical interaction between these two proteins (Fig. 6d). Furthermore, we conducted reciprocal co-immunoprecipitation (Co-IP) assays to validate this finding. The results demonstrated an interaction between these two molecules in LECs (Fig. 6e). Additionally, overexpression of EphrinB2 further enhanced the interaction between ISL1 and CDK5, leading to increased ISL1 phosphorylation in LECs under hypoxic conditions (Fig. 6f). Then, we investigated whether EphrinB2-induced ISL1 nuclear translocation was CDK5-dependent. Accordingly, we constructed Adv-sh*CDK5* and Ad-shScram vectors, and the knockdown efficiency of Adv-sh*CDK5* was confirmed by Western blotting (supplementary Fig 12a, b). As a consequence of inhibited CDK5 activity, lower phosphorylation levels of ISL1 at Serine/Threonine(S/T) residues were observed in pulldown protein lysates in Ad-*Efnb2* transfected LECs under hypoxia, while the overall protein expression of ISL1 remained comparable between Adv-sh*CDK5* transfected and non-transfected LECs (Fig. 6g). Meanwhile, we observed that EphrinB2-induced nuclear accumulation of ISL1 was abolished by inhibiting CDK5 in LECs under hypoxia (Fig. 6h). Furthermore, CDK5 silencing disrupted EphrinB2-mediated LEC migration and proliferation in vitro, consistent with the effect of ISL1 knockdown (Fig. 6i–k). The data indicated that EphrinB2 facilitated the nuclear translocation of ISL1 by stimulating the CDK5-dependent phosphorylation process, ultimately contributing to the promotion of lymphangiogenesis.

### VEGFR3 inhibitor MAZ51 disrupts EphrinB2-mediated lymphangiogenesis

To demonstrate the functions of EphrinB2 through the CDK5/ISL1/VEGFR3 signaling pathway in enhancing lymphangiogenesis and exerting cardioprotective effects after acute myocardial infarction (MI), the VEGFR3 inhibitor MAZ51 was applied in vivo (supplementary Fig 13a). First, we treated MI mice with MAZ51. We found that the improvement in cardiac function with EphrinB2 overexpression was significantly diminished by MAZ51 (supplementary Fig 13b–e). VEGFR3^+^ lymphatic vessels were significantly reduced in MAZ51-treated MI mice following EphrinB2 overexpression (supplementary Fig 13f, g). Furthermore, we treated sh*CDK5*-knockdown LECs with MAZ51. Consistent with previous results, silencing *CDK5* suppressed EphrinB2-mediated proliferation and migration in LECs. Furthermore, MAZ51 treatment produced similar inhibitory effects as Adv-sh*CDK5* following EphrinB2 overexpression in LECs (supplementary Fig 13h–j). Notably, MAZ51 did not further affect LEC proliferation and migration after CDK5 knockdown. These data further demonstrated that EphrinB2 through the CDK5/VEGFR3 signaling pathway mediated lymphangiogenesis and improved cardiac function after MI.

## Discussion

We uncovered direct evidence that EphrinB2, a receptor tyrosine kinase (RTK) on the cell membrane of LECs, plays a key role in regulating lymphangiogenesis after MI. We observed that EphrinB2 deficiency was associated with significant impairment of cardiac structure and function following acute MI. EphrinB2 overexpression enhanced cardiac lymphangiogenesis, accelerated inflammation resolution, and improved cardiac function after MI. Mechanistically, EphrinB2 enhanced the transcriptional activity of ISL1 by activating the CDK5 pathway. Phosphorylation of ISL1 in a CDK5-dependent manner promoted its nuclear translocation, facilitating the transcription of the FLT4 gene, which encodes the lymphatic-specific receptor VEGFR3. The CDK5/ISL1/VEGFR3 signaling pathway, downstream of EphrinB2, ultimately resulted in enhanced migration and proliferation of LECs, promoting lymphangiogenesis and reducing cardiac macrophage accumulation and inflammation in vivo. Our work extends the prior observations that VEGFC-mediated LEC activation induced cardiac lymphangiogenesis after MI. This study may provide a new therapeutic avenue for ischemic heart disease by regulating EphrinB2-mediated cardiac lymphangiogenesis.

The lymphatic system is crucial for maintaining the structural and functional integrity of the heart under both physiological and pathological conditions. The beneficial effects of lymphangiogenesis after MI have been extensively studied, particularly its role in reducing post-MI myocardial inflammation and edema^28^. The VEGF-C/VEGFR-3 axis is the most studied target for improving cardiac function through lymphangiogenesis after MI^2,3^. VEGF-C is recognized as a potent stimulator of lymphangiogenesis; however, it also contributes to increased vascular permeability and the extravasation of lymph from lymphatics^29^. Our study identified EphrinB2 as an alternative stimulator of cardiac lymphangiogenesis after MI.. Moreover, we demonstrated that EphrinB2-driven lymphangiogenesis occurs through a novel mechanism involving the upregulation of ISL1. EphrinB2 has been extensively studied for its crucial role in angiogenesis, vasculogenesis, and lymphangiogenesis during embryonic development^8,30^. However, the role of EphrinB2 and its mechanism in regulating cardiac lymphangiogenesis and remodeling after pathological disease models, including myocardial infarction and heart failure, remain undemonstrated. We uncovered a novel role of EphrinB2-mediated lymphangiogenesis in regulating cardiac function following MI.

To uncover the molecular mechanisms by which EphrinB2 stimulated lymphangiogenesis, we studied its downstream signaling pathways following comprehensive RNA-seq analysis. We particularly focused on ISL1, a DEG involved in endothelial cell proliferation. ISL1 is a LIM-homeodomain transcription factor that was initially thought to bind to an enhancer of the insulin gene^31^. Furthermore, ISL1-expressing progenitors were identified as a potential non-venous source of cardiac LECs^18^. We observed that EphrinB2 substantially upregulated ISL1 expression at both the mRNA and protein levels under hypoxia. Meanwhile, knocking down ISL1 reduced EphrinB2-driven proliferation and migration of LECs, indicating a role of ISL1 downstream of EphrinB2. EphrinB2 overexpression promoted the phosphorylation of ISL1, facilitating its nuclear translocation and the subsequent transcription of VEGFR3 (*FLT4*) in a CDK5-dependent manner. Specifically, CDK5 interacted physically with and phosphorylated ISL1. Inhibiting CDK5 reduced the transcriptional activity of ISL1, resulting in limited EphrinB2-driven proliferation and migration of LECs. These results indicated an intriguing phenomenon: ISL1 not only plays a vital role in cardiac lymphatic vessel development but can also be reactivated in response to pathological stimuli. The reactivation of ISL1 is the essential molecular mechanism underlying EphrinB2-enhanced lymphangiogenesis. In this study, we further demonstrated that the VEGFR3 inhibitor MAZ51 disrupted EphrinB2-enhanced lymphangiogenesis, thus abolishing the cardioprotective effects of EphrinB2 on post-MI phenotypes. This indicates that EphrinB2 promoted lymphangiogenesis through VEGFR3 signaling. For the first time, we illustrated the link between the EphrinB2/CDK5/ISL1/VEGFR3 axis and cardiac lymphangiogenesis-mediated cardioprotection post-MI. These findings suggest a potential therapeutic avenue for targeting EphrinB2 and its downstream pathways to activate cardiac lymphangiogenesis. Notably, Su et al. demonstrated a profibrotic role for EphrinB2 in cardiac fibrosis^32^. They found that EphrinB2 overexpression promoted the conversion of fibroblasts to myofibroblasts, accompanied by enhanced migration and proliferation capabilities. The pro-migratory and pro-proliferative effects of EphrinB2 on fibroblasts and myofibroblasts illustrated in their study were consistent with our observations in LECs. However, the activation of EphrinB2 in fibroblasts or LECs, contributing to cardiac fibrosis or lymphangiogenesis, respectively, could lead to distinct post-MI cardiac phenotypes. Therefore, much caution is needed when performing cell-type selective targeting of EphrinB2 in the context of translational applications for cardiovascular disease. Additionally, unraveling the cell-type-dependent EphrinB2 signaling is crucial for developing targeted therapies to treat ischemic heart disease.

Cardiac lymphatic vessels are critical for both healthy and ischemic myocardium as they facilitate the transport of extravasated proteins and fluids out of the myocardium and promote the egress of inflammatory cells^33,34^. Numerous studies have shown that following myocardial infarction, the density of lymphatic capillaries around the infarct zone increases. However, the endogenous lymphangiogenic network appears unable to compensate for the homeostatic derangement of ischemic myocardium. In our study, we observed an accelerated resolution of inflammatory cells and mediators triggered by the EphrinB2 overexpression-mediated cardiac lymphangiogenic response after MI. Specifically, we found that EphrinB2 overexpression improved the drainage of CD45^+^CD11b^+^ Ly6G^−^ F4/80^+^ Ly6C^high^ macrophages from infarcted areas to the MLNs. The accumulation of macrophages was associated with the sparsity of lymphatic vessels in the injured myocardium. EphrinB2 overexpression enhanced the lymphangiogenic response, promoting inflammation resolution, and thereby facilitating post-MI repair. Previous studies reported that the deletion of LEC membrane-anchored LYVE1 disrupted the adhesion and transit of macrophages and dendritic cells via LECs, leaving the lymphatic structures unaffected^21,25^. Therefore, we utilized *Lyve1* knockout mice to determine the intrinsic lymphatic function in inflammation resolution following MI. We also observed that LYVE1 deficiency abrogated the EphrinB2-mediated cardioprotective effects due to defects in immune cell clearance through lymphatics after MI. In light of this evidence, we believe that EphrinB2-mediated lymphangiogenesis helps modulate the acute immune response and normalizes the potential long-term chronic inflammatory environment, thereby favoring better post-MI tissue repair and outcomes^18,31^. Therefore, approaches to enhance EphrinB2 signaling may represent a novel therapeutic avenue for treating myocardial ischemia by enhancing cardiac lymphangiogenesis in acute MI patients.

In summary, EphrinB2 overexpression effectively promotes cardiac lymphangiogenesis, alleviating cardiac inflammation by enhancing lymphatic drainage and reducing macrophage accumulation in the infarcted area. In terms of the molecular mechanism, EphrinB2 regulates the transcription of lymphatic VEGFR3 (*FLT4*) by facilitating the nuclear translocation of ISL1, which occurs upon CDK5-dependent phosphorylation. In our study, EphrinB2 overexpression was associated with a significant improvement in cardiac function and remodeling, serving as a potent stimulator of exogenous cardiac lymphangiogenesis and a potential therapeutic target.

## Materials and methods

### Animals

All animal study was approved by the Animal Ethics Committee of Fudan University (Shanghai, China). Wild-type, *Efnb2*^em1cyagen^ mice, and *Lyve1*^em1cyagen^ mice on a C57BL/6 background were purchased from the Cyagen Biosciences (Guangzhou, China), *Lyve1*-Cre; *Rosa26*-tdTomato mice were used in our previous study^35^. Exon7-9 of the *Efnb2* gene or Exon2-5 of the *Lyve1* gene were deleted in these mice by the CRISPR-cas9 method. The primers for mouse genotype identification are as follows, *Efnb2*^em1cyagen^ mice: forward primer: 5’-TGGGGCTTAGATTTCAGTCTCCTAC-3’, reverse primer: 5’-GTACACTGCCTATTTTCCTGCCTG-3’; *Lyve1*^em1cyagen^mice: forward primer: 5’-GGACCTGAAACGGAGAACTCAC-3’, reverse primer: 5’-CTCTTCCTTTACAACCTTGGTTTCG-3’. All mice were backcrossed onto a C57BL/6 background and bred for more than 6 generations. We used heterozygous *Efnb2*^em1cyagen^ (*Efnb2*^*+/−*^) mice as the homozygous *Efnb2*^em1cyagen^ (*Efnb2*^*−/−*^) mutation is lethal. All animals were housed in specific pathogen-free (SPF) conditions.

### Murine myocardial infarction (MI) models

Male, 8-10 weeks old mice (including WT, *Efnb2*^*+/−*^, *Lyve1*^*−/−*^ and *Lyve1*-Cre; *Rosa26*-tdTomato mice) were randomly assigned to sham or MI operation with a single sequence of simple randomization assignments. Briefly, mice were placed on the heating board in the supine position when anesthetized by inhalation with 2-3% isoflurane. After making a small skin incision (around 1 cm), dissecting the pectoral major and minor muscle, and entering the chest at the fourth intercostal space using mosquito forceps, the heart was manually externalized. Next, a 6-0 silk suture was used to penetrate the myocardium to permanently ligate the left anterior descending artery (LAD). Effective LAD ligation was confirmed by the pallor of the left ventricle (LV) and ST-segment elevation by electrocardiography. Subsequently, the heart was returned to the original cavity and the skin was sutured. The sham operation did not include the ligation of LAD. The experimental protocols were approved by the Institutional Animal Care and Use Committee of Fudan University and investigators followed the ethical code of animal use.

### Cell extract and fluorescence-activated cell sorter (FACS)

We prepared the single-cell suspension from post-MI hearts as previously described^36^. Briefly, we immediately harvested murine hearts and cut them into small pieces. The samples were enzymatically digested with type II collagenase (1.5 mg mL^−1^, Worthington Biochemical Corporation, Lakewood, NJ, USA), DNase I (0.5 mg mL^−1^, Worthington Biochemical Corporation), and elastase (0.25 mg mL^−1^, Worthington Biochemical Corporation)in a shaker for 40 minutes at 37 °C. Next, the tissue suspension was gently filtered through a 70-μm cell strainer. We utilized fixable viability stain 510 (1:1000; #564406, BD Biosciences) as an indicator of cell viability before staining with primary antibodies for fluorescence-activated cell sorter (FACS) analysis. The antibodies used for FACS were as follows: CD45-PE (1:200; #553081, BD Biosciences), CD11b-PE-Cy7 (1:200; #552850, BD Biosciences), Ly6G-FITC (1:200; #551460, BD Biosciences), Ly6C-APC(1:200; #17-5932-82, Invitrogen), F4/80-BV421(1:200; #565411, BD Biosciences) at 4 °C for 30 min. The results were expressed as the percentage relative to CD45-positive cells. FACS was performed with a FACSAria™ flow cytometer (BD Biosciences). The data were further analyzed with FlowJo 10.8.1 (Tree Star Inc., Ashland, USA).

### Transthoracic echocardiography analysis

Echocardiography was conducted on mice after MI or sham operation by a Vevo2100 VisualSonics ultrasound system (VisualSonics Inc., Toronto, Canada) with an MS-400 imaging transducer as described previously^37^. First, mice were placed in a supine position when anesthetized with inhalation of 1.0-2.0% isoflurane. The heart rate was kept between 400 bpm and 600 bpm. B-mode images were acquired in the LV long-axis plane while M-mode images were at the mid-papillary muscle level. All the measurements were double-blind. LV function was analyzed offline using VevoLAB software (FUJIFILM VisualSonics). LV internal diameters during end-systole (LVID;s) or end-diastole (LVID;d), and ejection fraction (EF) were calculated by the software.

### Histology

When the mouse was euthanized under deep anesthesia, their chest was opened and the heart was exposed to the air, and the perfusion needle was inserted in the apex of the heart to rapidly perfuse sterile saline followed by fixation of the excised heart with 4% paraformaldehyde (PFA) overnight. Heart tissues were dehydrated with 30% sucrose overnight followed by being embedded in paraffin, and the fixed hearts were longitudinally sliced into 5 μm-thick sections with an interval of 200 μm. Alternatively, we embedded fresh heart tissues into an optimum cutting temperature (OCT) compound (Sakura, Torrance, CA, USA) and frozen them at −80 °C. Frozen hearts were cryosectioned into 10 μm-thick transverse sections at different levels. The cryosections were stored at −80 °C for further staining.

### Evaluation of infarct size and infarct wall thickness

The longitudinal tissue sections were stained with hematoxylin and eosin (HE) and Masson’s trichrome to evaluate the infarct size and wall thickness. Serial heart longitudinal sections were recorded and acquired using a microscope (Leica, Wetzlar, Germany). Myocardial infarct size was measured as the proportion of total infarct circumference to total LV circumference, as described previously^36^. The data were analyzed by Fiji (National Institutes of Health, Bethesda, MD, USA).

### Tissue clearing protocol

The hearts of lymphatic reporter mice (*Lyve1*-Cre; *Rosa26*-tdTomato) were perfused with phosphate-buffered saline (PBS) and a 4% PFA solution. The samples were incubated in 4% PFA at 4 °C in a shaker overnight. Next, the fixed samples were rinsed with PBS for 2 hours twice at room temperature (RT). The clearing protocol was conducted with the Nuohai Tissue Clearing Kit (Nuohai Life Science (Shanghai) Co., Ltd, Shanghai, China). Next, the samples were incubated in 20 mL Solution 1 for 7 days within a shaker at 37 °C, and the clearing solution was refreshed every day. Then each sample was rinsed with PBS for 6 hours in a shaker at RT and the PBS was refreshed for 2 hours. Equal volume Solution 2 was used to immerse each sample for another 7 days at 37 °C. Finally, the samples were RI matched with Solution 3 for 3-dimensional (3D) tissue imaging.

### 3D tissue imaging and image processing

3D tissue imaging of the cleared tissues was conducted with a Nuohai LS 18 Tiling Light Sheet Microscope (Nuohai Life Science (Shanghai) Co., Ltd, laser lines: 405, 488, 561, 637 nm). A 6-tile tiling light sheet was used to illuminate the sample^38^, and a 1×/0.25NA objective (Olympus MVPLAPO) was utilized to collect the signal. The magnification of the microscope was set at 6.3×, and the spatial resolution was roughly 2 × 2 × 5 µm3 at the selected imaging conditions. The collected images were integrated using the LS 18 ImageCombine software (Nuohai Life Science (Shanghai) Co., Ltd) and rendered with Amira (Thermo Fisher Scientific, USA).

### Immunofluorescence staining

The cryosections were dried at RT for 1 hour, fixed by 4% PFA for 10 minutes, and then rinsed with PBS three times. Next, the fixed cryosections were permeabilized with 0.3% Triton X-100 (Beyotime, China) for 10 minutes, blocked in PBS containing 3% bovine serum albumin (BSA) for 1 hour at RT, and incubated with primary antibodies overnight at 4 °C in a humidified chamber. The antibodies utilized were as follows: EphrinB2 (10 μg/ml;# AF496, R&D system), VEGFR3/FLT4 (2 μg/ml; #AF743, R&D system), LYVE-1 (1:100; #67538, Cell Signal Technology), CD31 (1:100; #ab9498, Abcam), CD68 (1:100; #25747, Proteintech), cTnT (1:100; #ab8295 Abcam), αSMA (1:500; #ab7817, Abcam), ISL1(1:200; #ab86501, Abcam), CDK5 (1:200; #10430-1-AP, Proteintech). Afterward, the cryosections were rinsed with PBS three times each for 5 min and incubated with Alexa fluorescence-conjugated secondary antibodies (Invitrogen, Carlsbad, CA, USA) for 1 hour at 37 °C. Then the slides were rinsed with PBS again three times and finally incubated with 4'6-diamidino-2-phenylindole (DAPI) (Beyotime, Shanghai, China) at RT for 5 min. Images were visualized using a fluorescence microscope (Leica, Wetzlar, Germany). VEGFR3^+^ vascular structures were considered VEGFR3^+^ lymphatics.

### Terminal deoxynucleotidyl transferase-mediated dUTP-biotin nick-end labeling (TUNEL) assay

Cell apoptosis was determined in sections by TUNEL assay (Beyotime, China), following the manufacturer’s instructions. First, the cryosections were fixed by 4% PFA for 10 minutes. Subsequently, the cryosections were incubated with a TUNEL working solution for 1 h at RT. Finally, the sections were incubated with 4'6-diamidino-2-phenylindole (DAPI) (Beyotime, China) for 5 mins. The images were obtained with a fluorescence microscope (Leica, Wetzlar, Germany). The proportion of apoptotic cells was measured as the percentage of TUNEL-positive nucleus number out of a total number of nuclei identified by DAPI staining.

### Western blotting analysis

Total protein was extracted from cardiac tissues or cultured cells with radioimmunoprecipitation assay (RIPA) buffer (Beyotime, Shanghai, China) containing phenylmethanesulfonyl fluoride (PMSF). Tissue lysates were then centrifuged at 13000 *g* and 4 °C for 15 min. Next, the supernatant was collected and the concentration of total protein was quantitated with the BCA working reagent (Beyotime, China). Next, each protein sample was adjusted to the same concentration by dilution with 5× loading buffer and boiled for 5 minutes at 95 °C. The protein lysates were separated on 10% or 12.5% sodium dodecyl sulfate-polyacrylamide gel electrophoresis (SDS-PAGE) gels by electrophoresis and transferred onto polyvinylidene fluoride (PVDF) membranes (Millipore, USA). The PVDF membranes were blocked in TBST with 5% bovine serum albumin (BSA) for 1 hour at RT and incubated with primary antibodies overnight at 4 °C. The antibodies utilized in this study were as follows: EphrinB2 (1 μg/ml; AF496, R&D system), VEGFR3/FLT4 (0.1 μg/ml; AF743, R&D system), LYVE1 (0.25 μg/ml; AF2125, R&D system), ISL1 (1:1000; ab86501, Abcam), CDK5 (1:2000; ab40773, Abcam), Pan phosphor-Serine/Threonine (1:1000; AP1745, ABclonal), β-actin (1:5000; KC-5A08, KangChen). Finally, The PVDF membranes were rinsed in TBST for three times and incubated with HRP-conjugated secondary antibodies for 1 hour RT. The specific bands were detected by ChemiDoc Touch Imaging System (Bio-Rad, CA, U.S.A.) after interacting with ECL western blotting substrate (Tanon, Shanghai, China). Data were analyzed using the Image Lab software (Bio-Rad, CA, U.S.A.).

### Immunoprecipitation and immunoblot analysis

Total protein from whole lysates was immunoprecipitated using primary antibodies. The extract was incubated for 12 h at 4 °C with an anti-ISL1 or CDK5 antibody followed by the addition of protein-A/G agarose beads and then incubated for 8 hours at 4 °C. The beads were centrifuged at 2500 rpm for 5 minutes and rinsed with PBS for 3 times. Subsequently, the beads were re-suspended with protein loading buffer and boiled at 95 °C for 5 minutes. The supernatant was collected to for immunoblotting. Anti-Pan Phospho-Serine/Threonine, ISL1, and CDK5 antibody were used respectively to detect immunoreactive bands for 12 hours at 4 °C and detected by ChemiDoc Touch Imaging System (Bio-Rad, CA, U.S.A.).

### Adeno-associated virus serotype vectors (AAV) construction and injection

For ephrinB2 overexpression in vivo, a full-length sequence of *Efnb2* (GenBank ID: NM_010111.6) was cloned into an AAV plasmid, and then the plasmid was subcloned into the AAV vector. An empty AAV vector was constructed as a negative control (NC). 6-week-old mice were injected with 100 μl of recombinant pAAV plasmid (1 × 10^11^ TU/ ml) via tail-vein injection. For ephrinB2 knockdown in vivo, the RNA interference (RNAi) sequence targeting mouse *Efnb2* was 5’-GCTAGAAGCTGGTACAAAT-3’. An empty AAV vector was generated as a negative control. One month after infection, some of the mice were sacrificed and the overexpression or knockdown of the EphrinB2 protein was confirmed by western blotting.

### RNA extraction and RT-qPCR

Total RNA was extracted from heart homogenate using UNIQ-10 Column Trizol Total RNA Isolation Kit (Sangon Biotech, China) according to the manufacturer’s protocol. Next, cDNA was synthesized from RNA samples using PrimeScript^TM^ RT Master Mix (TaKaRa, Japan). RT-PCR was conducted using qPCR SYBR Master Mix (Vazyme Biotech, Nanjing, China), and PCR reactions were performed in an S1000 PCR System (Bio-Rad, CA, U.S.A.) at an initial 94 °C for 1.5 min and then 30 cycles of 94 °C for 20 s, 56 °C for 20 s, 72 °C for 60 s, and a final extension at 72 °C for 5 min. *18* *s* was used as an internal control. The relative mRNA levels of genes were quantified using the comparative ∆∆CT method, normalized to *18S*, and presented relative to a control group.

### Chromatin immunoprecipitation assay

Once cells reached above 90% confluent, a 1% formaldehyde solution was added and incubated for 15 minutes at RT to facilitate the cross-linking of proteins to genome DNA. Following this, glycine buffer was introduced to halt any additional cross-linking. According to the instructions of SimpleChIP® Enzymatic Chromatin IP Kit (Agarose Beads) (Cell Signaling Technology, U.S.A.), cells were then lysed, and chromatin was extracted and fragmented through both enzymatic digestion and sonication. Subsequently, the resulting DNA/protein fragments underwent immunoprecipitation with an anti-ISL1 antibody. The immunoprecipitated complex was then enriched with ChIP Grade Protein A/G Plus Agarose. Subsequently, the immunoprecipitated chromatin was eluted from the antibody/Protein A/G Plus Agarose, allowing for the reversal of protein-DNA cross-links. The *FLT4* promoter was detected using the forward primer 5′-TCGCTTGGTTACCTAGTTTCCCCA-3′ and reverse primer 5′-CCAAAGCCCTTTCATCCTCGCC-3’. According to the ISMARA database, the predicted ISL1 binding site on the *FLT4* promotor is 5’-CTTAATA-3’.

### Adenovirus construction and transfection

The *Efnb2* gene (GenBank ID: NM_010111.6) cDNA was cloned and inserted into the pDC315 vector (purchased from Hanbio Co. Ltd, Shanghai, China). Adv-*Efnb2* and Adv-NC were transfected into HEK293 cells. The adenoviruses in the HEK293 cells were purified and the viral titer was determined by plaque assays. The concentrations of Adv-*Efnb2* and Adv-NC were 1 × 10^10^ plaque formation unit (PFU)/ml, respectively. For *ISL1* knockdown in vitro, siRNA sequence (sense: GAGACAUGGUGGUUUAtt; antisense: UUUCUCCUUGCACCUCtt) was for producing shISL1 plasmid. For *CDK5* knockdown in vitro, forward (5’-3’): GGGAGATCTGCCTACTCAA; Reverse (5’-3’): TTGAGTAGGCAGATCTCCC was for producing sh*CDK5* plasmid. The construction and transfection of adenovirus with sh*ISL1* or scrambled sequence were produced.

The lymphatic endothelial cells (LECs) were transfected with adenovirus for 12 hours, followed by the replacement of a cultured medium.

### The hypoxia model for human lymphatic endothelial cells (hLECs)

hLECs^35^ were cultured using DMEM media (Thermo Fisher Scientific, U.S.A) without glucose and FBS, and incubated in a hypoxia incubator with oxygen concentration lower than 0.1% for 0.5-24 h. Then, cells were removed from the hypoxia incubator and harvested for analysis.

### CCK-8 proliferation assay

The cells were seeded in 96-well plates at a density of 10^5^/ml for each well and cultured in DMEM (Thermo Fisher Scientific, U.S.A) at a 37 °C incubator. After hypoxia for 0.5, 2, 6, and 24 hours, cells in each well were incubated with 20 μl CCK-8 assay reagent (Beyotime, Shanghai, China) for 40 minutes, according the manufacturer’s instructions. Optical Density (OD) was measured using a microplate reader at 450 nm.

### Scratch wound assay

In vitro, cultured LECs were first transfected by recombinant adenovirus encoding ephrinB2 (0.5 × 10^8^ PFU /well) or NC for 36 hours. For each well, 1 × 10^6^ cells were plated into a 6-well plate and cultured to reach above 90% confluence. Then the culture media was removed, and cells were scratched with a single stroke of a 20 μl pipette tip to make a wound. Then the cells were cultured in DMEM (Thermo Fisher Scientific, U.S.A) at 37 °C incubator under the condition of hypoxia or normoxia for 24 hours. The scratch wound healing was photographed and recorded.

### Bulk RNA sequencing (RNA-seq)

Total RNA was extracted from LECs transfected with Adv-NC or Adv-*Efnb2* using TRIzol Reagent. Next, RNA was reverse-transcribed and amplified according to the manufacturer’s instructions. Sequencing libraries were built on the Illumina Novaseq 6000 platform (Genedenovo, Guangzhou, China). First, we removed reads containing sequencing adapters or reads of poor quality in raw data. Second, transcriptional reads were mapped to the human genome (GRCh38) using HISAT2. For a threshold of log_2_ fold change ≥2.0 and adjusted *P* < 0.05, the differentially expressed genes (DEGs) were identified using DESeq2. The RNA-seq analysis were performed by Genedenovo, using standard and consistent procedures. For functional analysis, KEGG or GO enrichment analysis was performed using the R package of clusterProfiler.

### Structure-based docking model analysis between ISL1 and CDK5

The protein structure of ISL1 was predicted by the SWISS-MODEL (www.swissmodel.expasy.org) with Protein Data Bank (PDB) structure 4JCJ, chain B (sequence identity, 96.99%) as the template. The experimental structure of CDK5 was obtained from the PDB database (PDB ID:4AUB, chain A). Structures of proteins were uploaded to the ClusPro (https://cluspro.org/help.php) to predict their docking model. The resulting docking model was visualized by the PyMol (open-source version).

### Statistical analysis

Data were presented as mean ± standard error of the mean (SEM) or standard deviation (SD). The number of biological replicates (n) is indicated in each figure legend. All statistical analyses were conducted using GraphPad Prism 8.0 software (GraphPad Software, Inc., La Jolla, CA) or R 4.0.1 software (R Foundation for Statistical Computing, Vienna, Austria). Normality of the data was determined by the Shapiro-Wilk test. For data that satisfied the normality assumption, we used with two-tailed unpaired Student’s test for the comparison between two groups or one-way or two-way repeated measurement ANOVA with Tukey post-hoc test for multiple group comparisons. Otherwise, we analyzed with the non-parametric Mann–Whitney *U* test for the comparison between two groups or the Kruskal–Wallis with Dunn test for multiple group comparisons. The correlation analysis was performed using the ggplot2 package in R software. Statistical significance was considered at *P < 0.05, **P < 0.01, ***P < 0.001, and ****P < 0.0001.

## Supplementary information

Supplementary Materials

3D Fluorescent Labeling of Mouse Cardiac Lymphatic Vessels: Myocardial Infarction (MI) Group

3D Fluorescent Labeling of Mouse Cardiac Lymphatic Vessels: Control Group

## Data Availability

The accession number for the RNA-seq data reported in this paper is GSE279328. Any additional information will be available from the lead contact upon request.
