# Supplementary material for: EphrinB2-mediated CDK5/ISL1 pathway enhances cardiac lymphangiogenesis and alleviates ischemic injury by resolving post-MI inflammation
Source: Signal Transduct Target Ther. 2024 Nov 18;9:326. doi: 10.1038/s41392-024-02019-4 (PMC11574162; doi:10.1038/s41392-024-02019-4)
Supplement: Supplementary file 1 — Supplementary Materials [file 41392_2024_2019_MOESM1_ESM.docx]

**Supplementary Materials For**

**The ephrinB2-mediated CDK5/ISL1 pathway enhances cardiac lymphangiogenesis and alleviates Ischemic injury by resolving post-MI inflammation.**

Yingnan Bai**^#,^** *, Liming Chen**^#^**, Fanghao Guo**^#^**, Jinghong Zhang, Jinlin Hu, Feixue Tao, Qing Lu, Wenyi Li, Xueying Chen, Ting Gong, Nan Qiu, Yawei Jin, Lifan Yang, Yu Lei, Chengchao Ruan, Qing Jing, John P. Cooke, Shijun Wang*, Yunzeng Zou*, Junbo Ge*

^#^ Yingnan Bai, Liming Chen, and Fanghao Guo contributed equally to this article.

*Corresponding authors: Yingnan Bai, MD & Ph.D. [bai.yingnan@zs-hospital.sh.cn](mailto:bai.yingnan@zs-hospital.sh.cn); Shijun Wang, [wang.shijun@zs-hospital.sh.cn](mailto:wang.shijun@zs-hospital.sh.cn); Yunzeng Zou, MD & Ph.D. [zou.yunzeng@zs-hospital.sh.cn](mailto:zou.yunzeng@zs-hospital.sh.cn); Junbo Ge, MD & Ph.D. [ge.junbo2@zs-hospital.sh.cn](mailto:ge.junbo2@zs-hospital.sh.cn).

**This PDF file includes:**

Figures. S1 to S13

**Other Supplementary Materials for this manuscript include the following:**

Movies S1 to S2**Figure. S1**

**
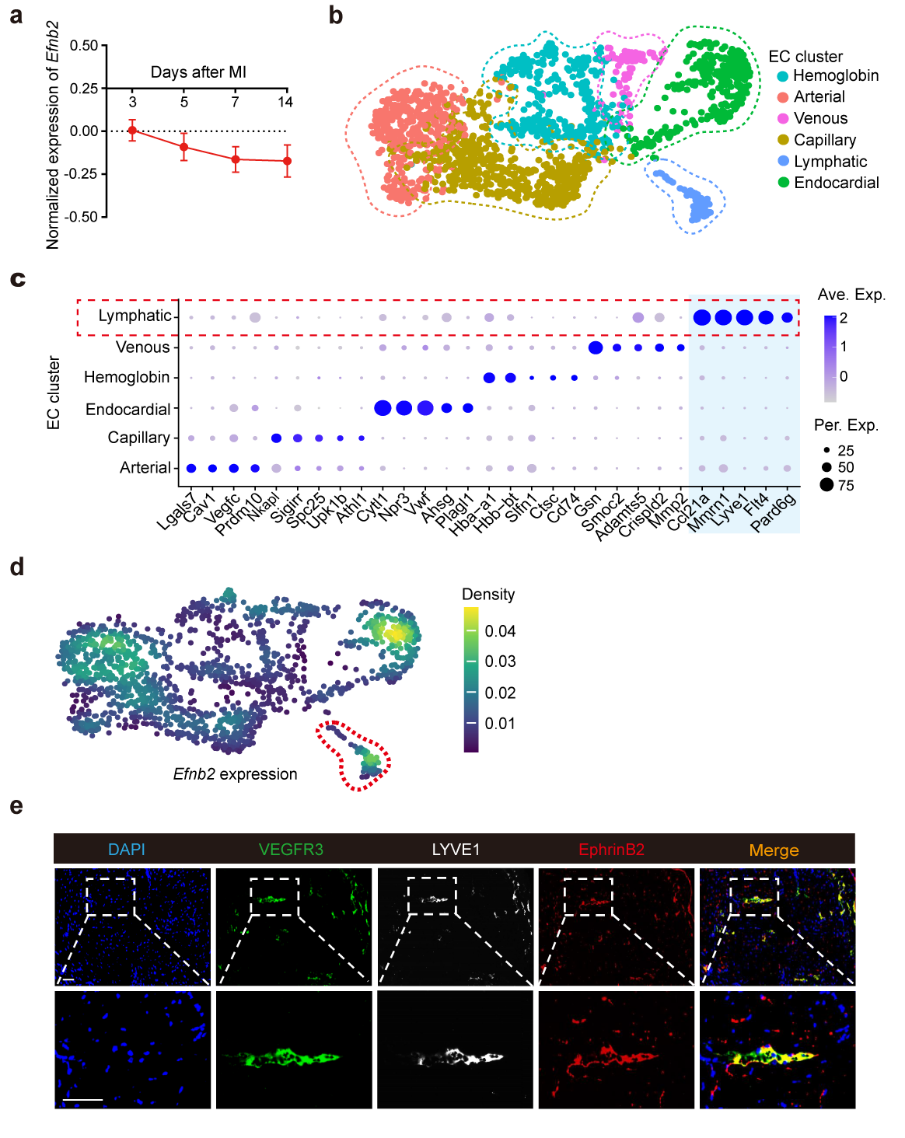
**

**a** The transcriptional expressions of *Efnb2* were examined in the microarray data (GSE6580) from mice subjected to MI surgery at four time points (3, 5, 7, and 14 days after MI) (n=5 per time point). **b** t-distributed stochastic neighbor embedding (t-SNE) plot showing the endothelial cells isolated from murine hearts clustered into six cell populations using single-cell RNA sequencing data (GSE120064). Colors denote different cell populations. **c** Dot plot displaying feature genes for the six-cell populations. **d** Feature plot showing the transcriptional expressions of *Efnb2* in each cell. Colors denote the relative expression of *Efnb2*. **e** Representative immunofluorescence staining images showing the myocardium co-stained by VEGFR3 (green), LYVE1 (white), EphrinB2 (red), and DAPI (blue). Magnified views of dashed white boxes are shown in the bottom lane. Scar bar: 20 μm. **P*<0.05, ***P*<0.01, ****P*<0.001, *****P*<0.0001. MI, myocardial infarction; and DAPI, 4′,6-diamidino-2-phenylindole.

**Figure. S2**


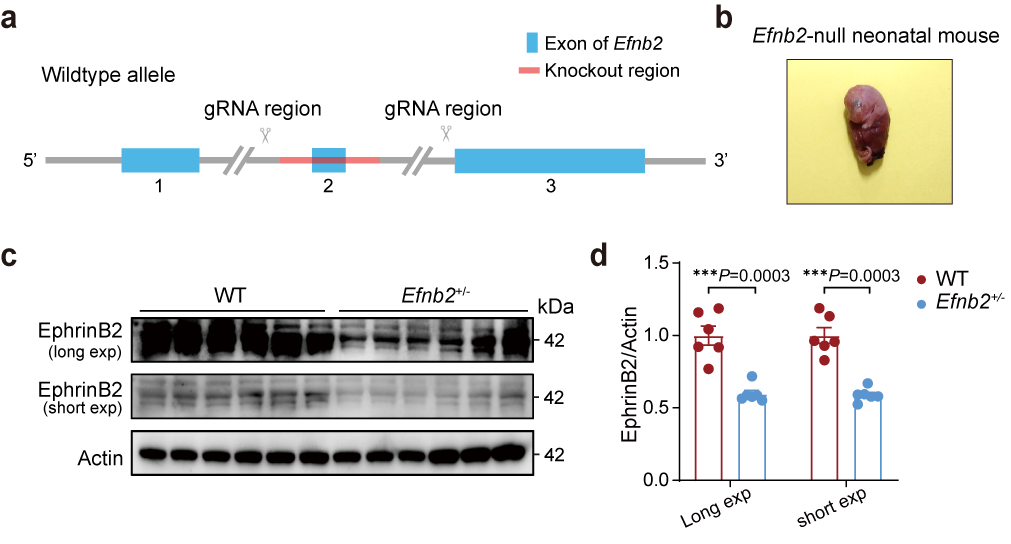


**a** The targeting strategy for constructing *Efnb2^+/-^* mice. **b** A representative image showing the *Efnb2*-null neonatal mouse. **c** Representative immunoblotting images showing EphrinB2 protein levels in hearts from *Efnb2*^+/-^ mice and their WT littermates. **d** Quantification of **c** normalized to Actin and presented relative to a sham group (n=6 per group). **P*<0.05, ***P*<0.01, ****P*<0.001, *****P*<0.0001. **d** by unpaired Student’s test. gRNA, guide RNA; long exp, long exposure; and short exp, short exposure.

**Figure. S3**

**
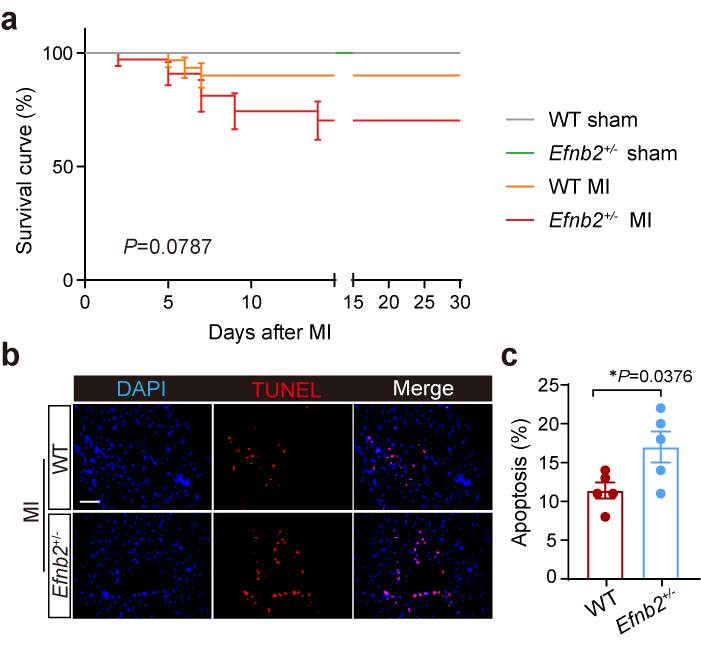
**

**a** Kaplan-Meier plot showing the survival probability of mice (n= 15,15,18,18 for the four groups, respectively). **b** Representative TUNEL staining images showing cell apoptosis in the myocardium from mice in indicated groups. Scar bar: 50 μm. **c** Quantification of **b**. (n=5 per group). **P*<0.05, ***P*<0.01, ****P*<0.001, *****P*<0.0001. **a** by log-rank test. **c** by unpaired Student’s test. TUNEL, terminal deoxynucleotidyltransferase-mediated dUTP-biotin nick end labeling.

**Figure. S4**

**
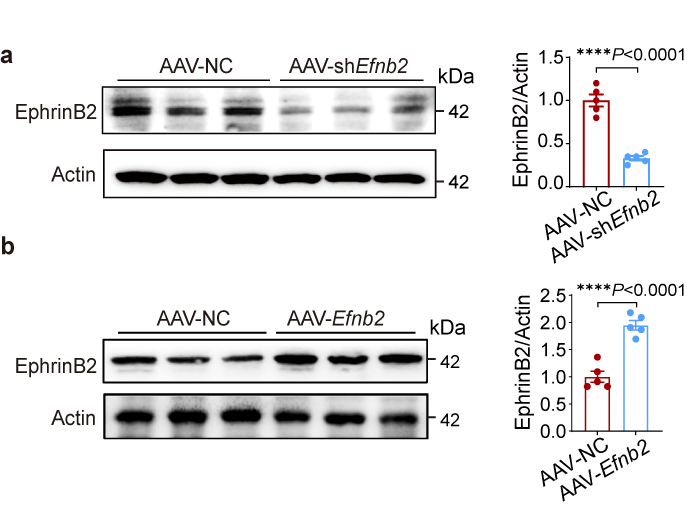
**

**a** (Left) Representative immunoblotting images showing EphrinB2 protein levels in the hearts of WT mice that were injected with AAV-NC or AAV-sh*Efnb2*. (Right) Quantification of immunoblotting results normalized to Actin and presented relative to a sham group (n=5 per group). **b** (Left) Representative immunoblotting images showing EphrinB2 protein levels in hearts of WT mice that were injected with AAV-NC or AAV-*Efnb2*. (Right) Quantification of immunoblotting results normalized to Actin and presented relative to a sham group (n=5 per group). **P*<0.05, ***P*<0.01, ****P*<0.001, *****P*<0.0001. **a, b** by unpaired Student’s test. AAV, Adeno-associated virus serotype. NC, negative control.

**Figure. S5**


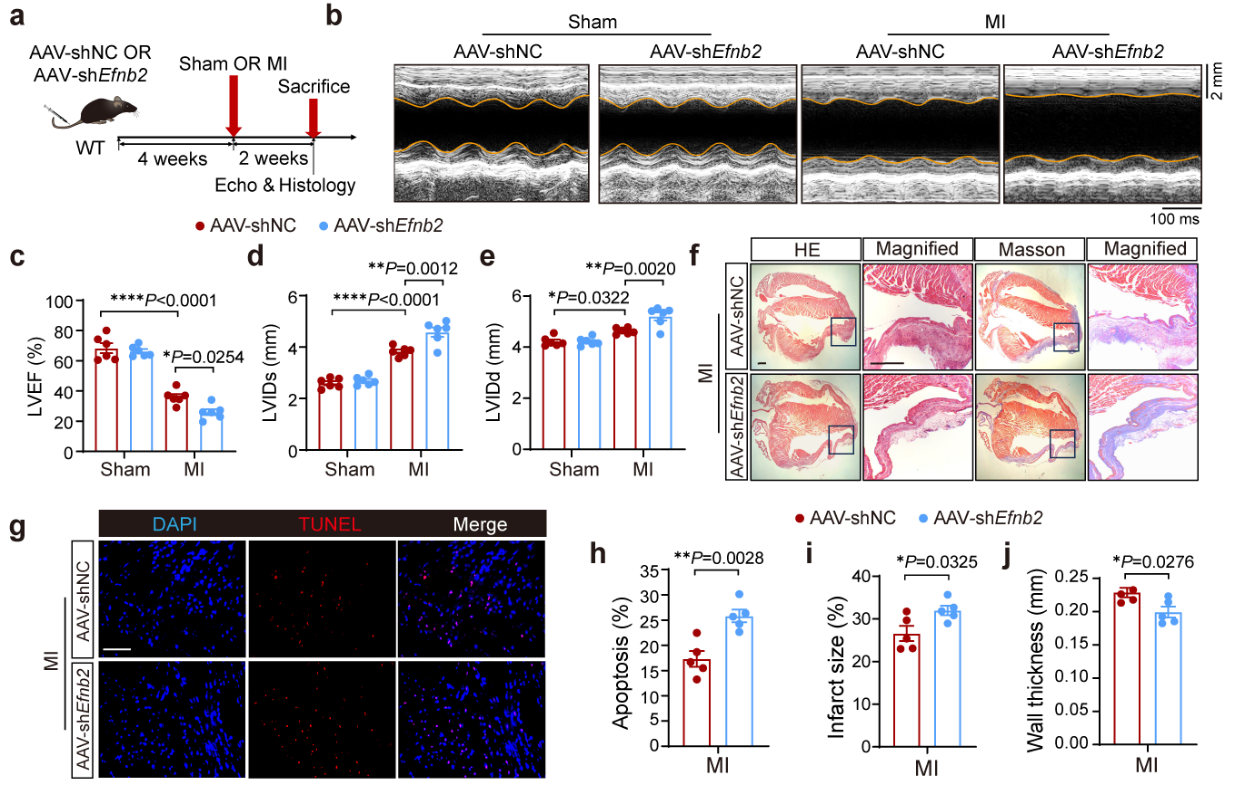


**a** Schematic diagram depicting the experimental strategy for EphrinB2 knockdown. **b** Representative M-mode echocardiographic images showing the cardiac function of WT mice that were injected with AAV-shNC or AAV-sh*Efnb2* after MI or sham operation. The yellow lines indicate the endocardium of the anterior and posterior walls at the mid-papillary level. **c-e** Quantification of echocardiographic parameters in **b** (LVEF, LVIDs, and LVIDd, n=6 per group). **f** Representative histological images showing infarct and fibrosis areas in the hearts of WT mice that were injected with AAV-shNC or AAV-sh*Efnb2* after MI operation assessed by HE and Masson Trichrome staining. Magnified views of black boxes are shown at the right of the gross views. Scar bar: 500 μm. **g** Representative TUNEL staining images showing cell apoptosis in the myocardium from mice in indicated groups. Scar bar: 50 μm. **h** Quantification of apoptotic cells in (g) (n=5 per group). **i, j** Quantification of infarct size of myocardium and wall thickness of infarct area in **a** (n=5 per group). **P*<0.05, ***P*<0.01, ****P*<0.001, *****P*<0.0001. **c-e** by one-way ANOVA with Tukey post-hoc test, and **h-j** by unpaired Student’s test. AAV, adeno-associated virus serotype. NC, negative control; HE, hematoxylin and eosin; LVEF, left ventricular ejection fraction; LVIDs, left ventricular internal dimension during systole; LVIDd, left ventricular internal dimension during diastole; DAPI, 4',6-diamidino-2-phenylindole; and TUNEL, terminal deoxynucleotidyltransferase-mediated dUTP-biotin nick end labeling.

**Figure. S6**

**
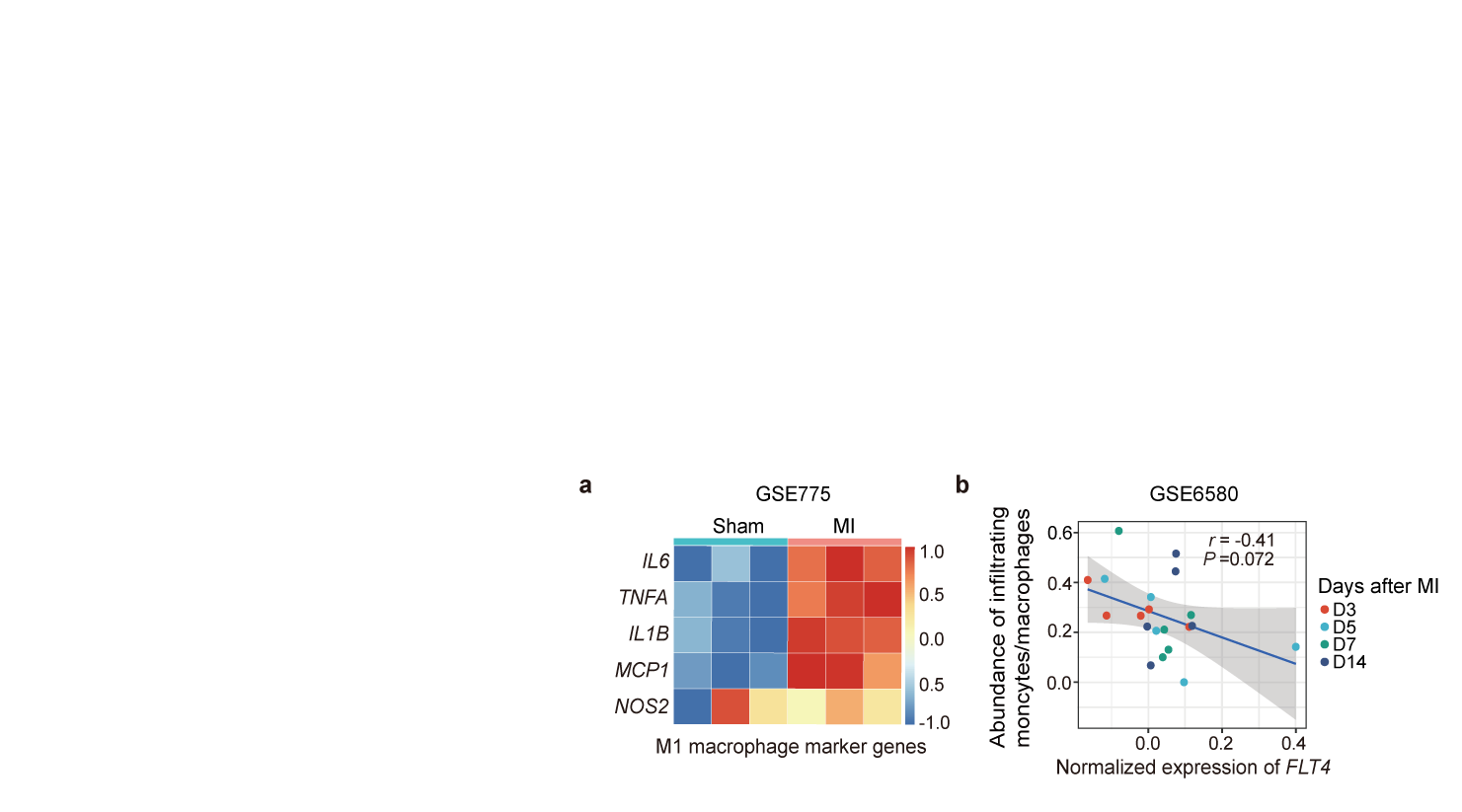
**

**a** The transcriptional levels of M1-type macrophage marker genes were examined using microarray data (GSE775) including mice subjected to MI. **b** Scatter plots depicting the correlation between the expression of *Flt4* and the abundance of infiltrated monocytes/macrophages in microarray data (GSE6580). **b** by Pearson correlation analysis. MI, myocardial infarction; D, days.

**Figure. S7**

**
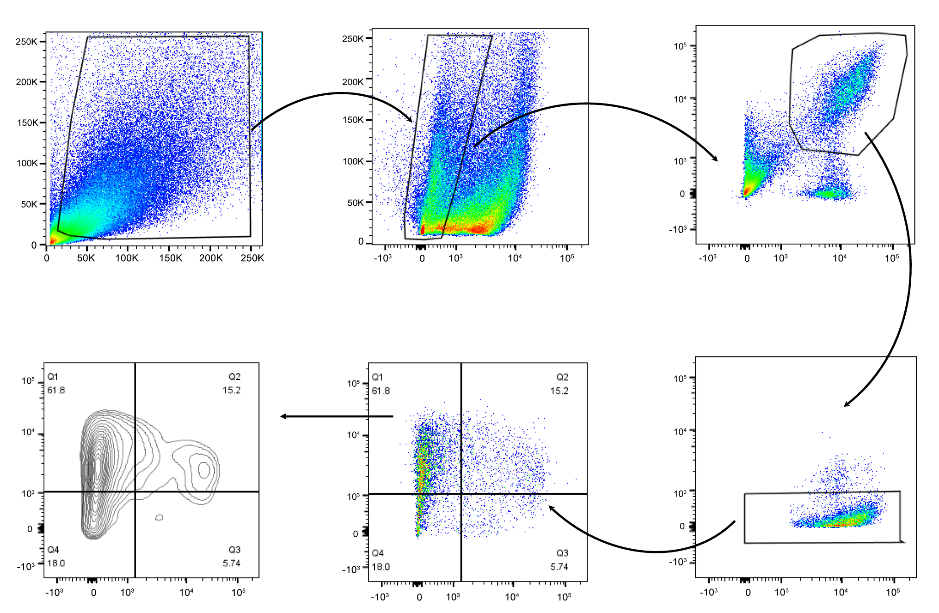
**

The gating strategy to sort the CD45^+^CD11b^+^ Ly6G^-^F4/80^+^Ly6C^high^ macrophages in the hearts of mice post-MI. SSC, side scatter; FSC, forward scatter; A, area.

**Figure. S8**

**
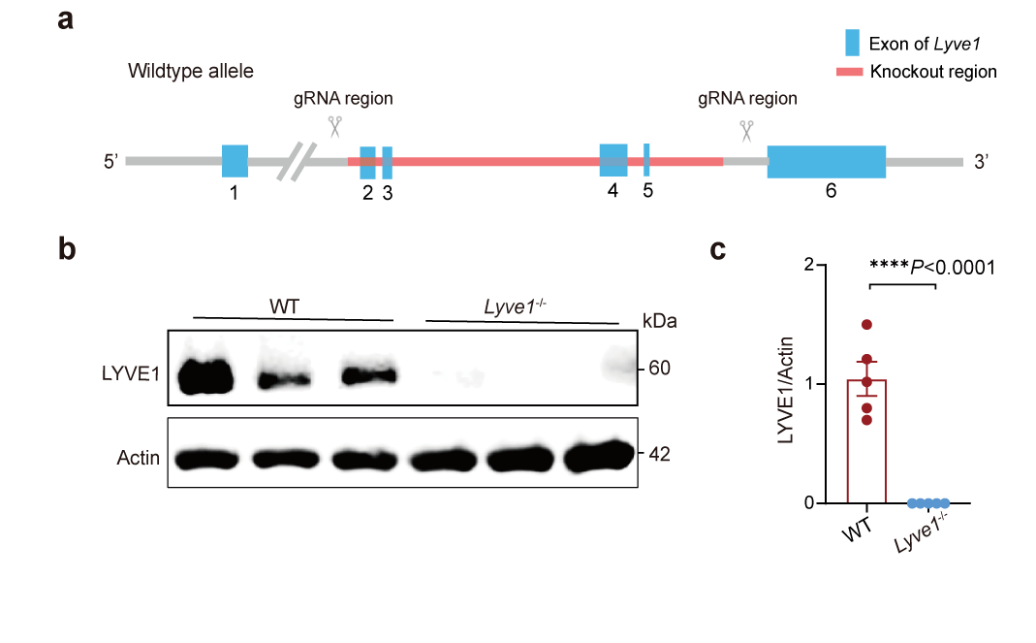
**

**a** The targeting strategy for constructing *Lyve1*^-/-^ mice. **b** Representative immunoblotting images showing LYVE1 protein levels in the hearts of *Lyve1*^-/-^ mice and their littermates. **c** Quantification of **b** normalized to Actin and presented relative to littermate controls (n=5 per group). **P*<0.05, ***P* <0.01, ****P*<0.001, *****P*<0.0001. **c** by unpaired Student’s test.

**Figure. S9**

**
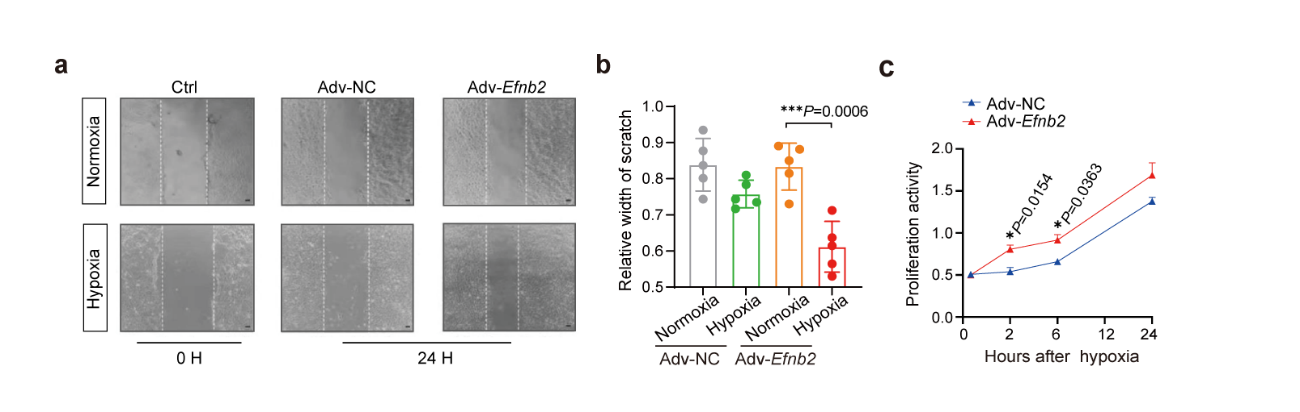
**

**a** Wound healing assay determining the effect of *Efnb2* overexpression under normoxic or hypoxic conditions for 24 hours. The white dashed lines indicate the terminals of the scratch. Scar bar: 100 μm. **b** Quantification of **a** presented relative to normoxia + Adv-NC group (n=5 per group). **c** Cell proliferation activity of LECs with Adv-NC or Adv-*Efnb2* under hypoxic conditions for indicated times (0, 6, 12, 24 hours) assessed by CCK-8 assay. **P*<0.05, ***P*<0.01, ****P*<0.001, *****P*<0.0001. **b** by one-way ANOVA with Tukey post-hoc test, **c** by one-way repeated measurement ANOVA.

**Figure. S10**

**
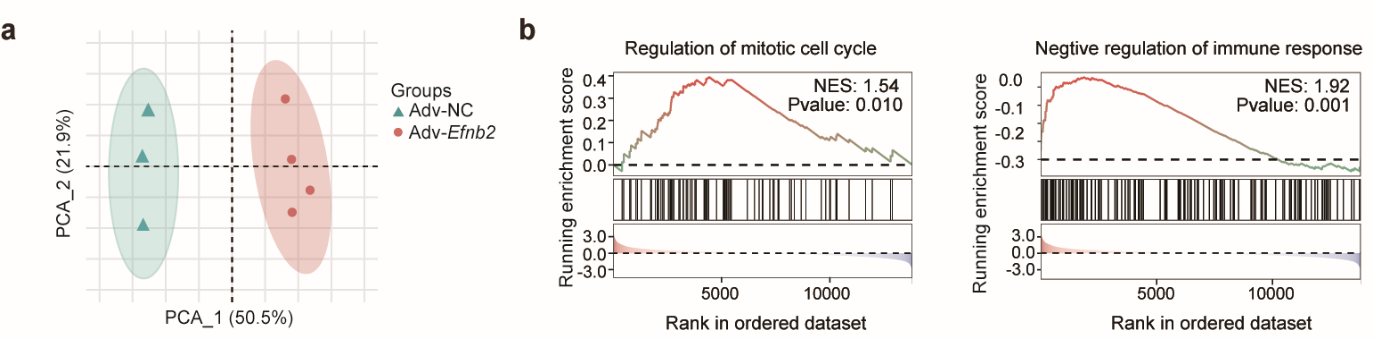
**

**a** Principal component analysis (PCA) showing the clustering of samples for LECs with Adv-NC or Adv-*Efnb2* under hypoxic conditions (n=3 for Adv-NC and 4 for Adv-*Efnb2* group). **b** Gene set enrichment analysis (GSEA) for key pathways.

**Figure. S11**


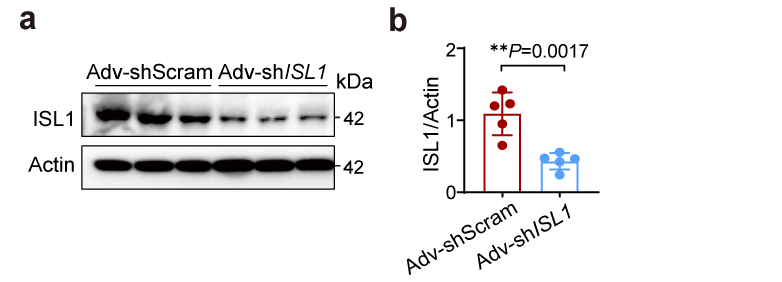


**a** Representative immunoblotting images showing ISL1 protein levels in LECs transfected with Adv- shScram or Adv-sh*ISL1*. **b** Quantification of **a** normalized to Actin and presented relative to Adv-shScram group (n=5 per group). **P*<0.05, ***P*<0.01, ****P*<0.001, *****P*<0.0001. **b** by unpaired Student’s test.

**Figure. S12**


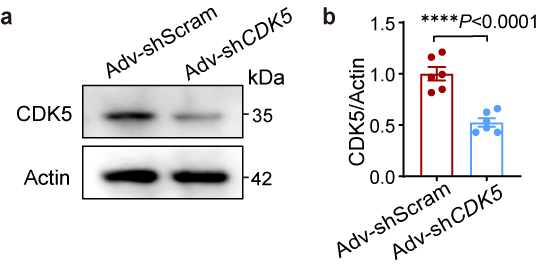


**a** Representative immunoblotting images showing CDK5 protein levels in LECs with Adv- shScram or Adv-sh*CDK5*. **b** Quantification of **a** normalized to Actin and presented relative to Adv-shScram group (n=6 per group). **P*<0.05, ***P*<0.01, ****P*<0.001, *****P*<0.0001. **b** by unpaired Student’s test.

**Figure. S13**

**
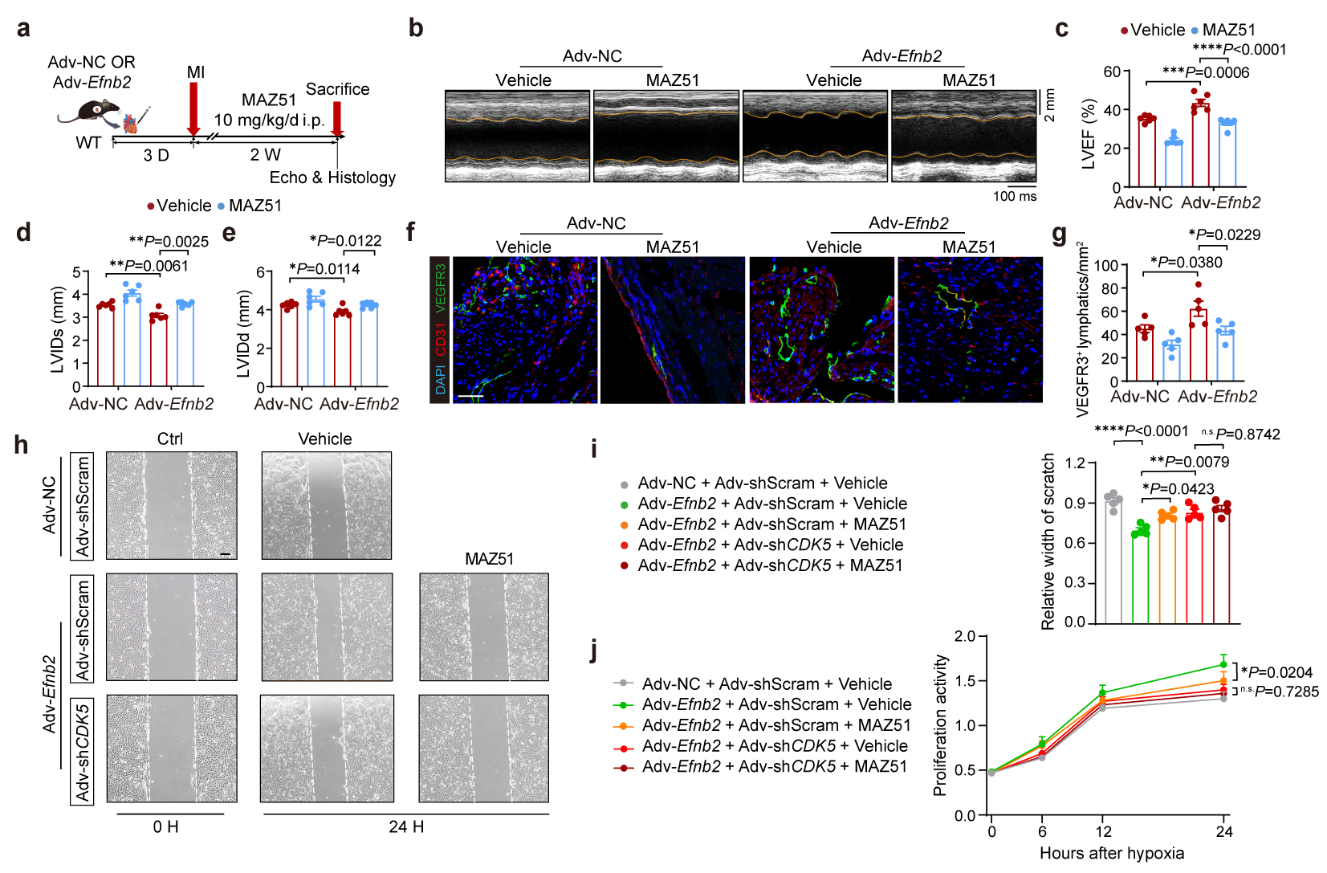
**

**a** Schematic diagram depicting the experimental strategy for EphrinB2 overexpression with MAZ51 treatment. **b** Representative M-mode echocardiographic images showing the cardiac function of WT mice in indicated groups after MI operation. The yellow lines indicate the endocardium of the anterior and posterior walls at mid-papillary levels. **c-e** Quantification of echocardiographic parameters in **b** (LVEF, LVIDs, and LVIDd, n=6 per group). **f** (Left) Representative immunofluorescence staining images showing the myocardium co-stained by CD31 (red), VEGFR3 (green), and DAPI (blue) of mice in indicated groups after MI operation. Scar bar: 50 μm. **g** Quantification of VEGFR3^+^ lymphatics (n=5 per group). **h** Wound healing assay determining the effect of MAZ51 on the LECs following *CDK5* knockdown under hypoxic conditions for 24 h. The white dashed lines indicate the terminals of the scratch. Scar bar: 100 μm. **i** Quantification of **h** presented relative to Adv-NC + Adv-shScram +Vehicle group (n=5 per group). **j** Cell proliferation activity of LECs in indicated groups under hypoxic conditions for indicated times (0, 6, 12, 24 hours) assessed by CCK-8 assay. **P*<0.05, ***P* < 0.01, ****P*<0.001, *****P*<0.0001. **c, d, e, g, and i** by one-way ANOVA with Tukey post-hoc test, and **j** by two-way repeated measurement ANOVA.
